# Pre-operative biomarkers and imaging tests as predictors of post-operative delirium in non-cardiac surgical patients: a systematic review

**DOI:** 10.1186/s12871-019-0693-y

**Published:** 2019-02-23

**Authors:** Farrah Ayob, Enoch Lam, George Ho, Frances Chung, Hossam El-Beheiry, Jean Wong

**Affiliations:** 1 Department of Anesthesia, Toronto Western Hospital, University Health Network, 2-434 McLaughlin Wing, 399 Bathurst Street, Toronto, ON M5T 2S8 Canada; 2 Faculty of Medicine, University of Toronto, 1 King’s College Circle, Medical Sciences Building, Room 2109, Toronto, ON M5S 1A8 Canada; 3 Department of Anesthesia, Trillium Health Partners, Mississauga Hospital, 100 Queensway, West, Mississauga, ON L5B 1B Canada; 4 Women’s College Hospital, Toronto, Ontario, 76 Grenville St, Toronto, ON M5S 1B2 Canada

**Keywords:** Systematic review, Predictive biomarkers and imaging, Predictive tests, Post-operative delirium, Predicting post-operative delirium

## Abstract

**Background:**

Post-operative delirium (POD) is a common post-operative complication in elderly individuals and imposes a significant health and financial burden. Identifying predictive biomarkers may help understand the pathophysiology of POD. Our objective is to summarize the evidence of pre-operative biomarkers and imaging tests to predict POD in patients undergoing non-cardiac surgery.

**Methods:**

A systematic search of English language articles in MEDLINE, EMBASE, Cochrane Database, PsychINFO, PubMed and ClinicalTrials. Gov up to January 2018 was performed. Studies that used biomarkers or imaging tests to predict POD and a validated POD assessment tool were included. Animal studies, paediatric, cardiac and intracranial surgery were excluded. Risk of bias was assessed using the Quality In Prognosis Study tool.

**Results:**

Thirty-four prospective cohort studies involving 4424 patients were included. Nineteen studies described serum tests [Interleukin-6, Insulin-like Growth Factor 1, C-Reactive Protein (CRP), cholinesterases, apolipoprotein-E genotype, leptin, hypovitaminosis, hypoalbuminaemia, gamma-amino butyric acid], 10 described cerebral-spinal fluid tests (monoamine precursor, melatonin, acute phase proteins, S100B and neurofibrillary tangles), and 5 described imaging tests. Two studies had high risk of bias due to unclear outcome measurement and study participation. CRP was significantly associated with POD in 5 studies. Other biomarkers were either examined by only a single study or two or more studies with conflicting results.

**Conclusion:**

CRP is the most promising biomarker associated with POD. However, we are still in the early stages in identifying biomarkers and imaging tests that may further understanding of the pathophysiology of POD.

**Electronic supplementary material:**

The online version of this article (10.1186/s12871-019-0693-y) contains supplementary material, which is available to authorized users.

## Background

Delirium is a common problem in hospitalized elderly individuals; it is defined as an acute onset of impaired cognitive functions, disturbance in attention and awareness that is not due to a pre-existing, established, or evolving neurocognitive disorder. Post-operative delirium (POD) usually emerges on post-operative day 1 to 3, after a lucid interval after emergence from anaesthesia [1]. The distinction between emergence agitation, post- anaesthesia care unit (PACU) delirium and POD are difficult to distinguish, their definitions have been based on review of literature and expert opinion [2]. Emergence agitation has been defined as agitation after discontinuation of inhaled anaesthetics, while PACU delirium has been defined as positive delirium signs after 30 min stay in PACU until discharge from PACU (delirium signs upon arrival in PACU may be considered continuation of emergence from general anaesthesia) [2]. PACU delirium may be a subset of POD and patients who experience PACU delirium are reported to have higher risk of POD [3].

The aetiology of POD is poorly understood but is probably due to the presence of underlying mechanisms of immune activation, oxidative stress and neurotransmitter imbalances in predisposed individuals [4]. Serious short and long-term adverse outcomes include persistent functional decline, prolonged hospital stay, higher morbidity and mortality, and increased health care costs.

Many delirium risk prediction models have low specificity [5]. There is increasing interest in investigating the pathogenesis of POD as reflected by the high number of studies examining various biomarkers. Investigating possible biomarkers to identify high-risk patients may advance understanding about the pathophysiology of delirium. This systematic review aims to summarize the current evidence with respect to biomarkers and imaging tests that may predict POD in non-cardiac surgery patients**.**

## Methods

### Search strategy

**Fig. 1:**
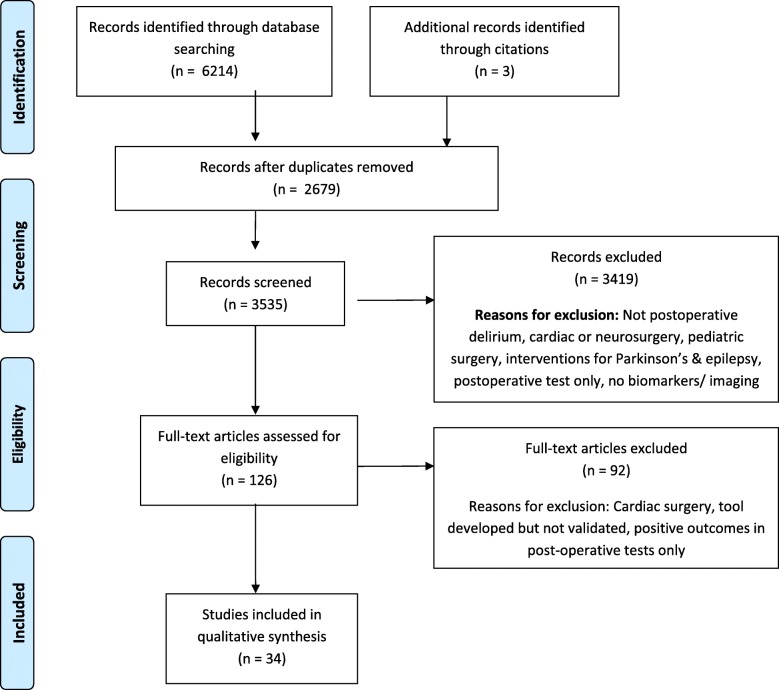
Flow diagram of study

A systematic search of the literature was performed according to Preferred Reporting Items for Systematic Reviews and Meta-Analyses statement (PRISMA) guidelines, Fig. 1. A comprehensive electronic search with the assistance of an expert medical librarian was conducted. The following databases were searched; MEDLINE (1946 to January 2018), Medline In-Process (up to January 2018), EMBASE (1947 to January 2018), Cochrane Central Register of Controlled Trials (up to December 2017), Cochrane Database of Systematic Reviews (2005 to January 2018), PsychINFO (1806 to January 2018), CINAHL, PubMed (up to January 2018) and ClinicalTrials. Gov (up to January 2018). We included randomized and non-randomized controlled trials, prospective and retrospective observational studies, and cross-sectional studies.

The search included a combination of the following MESH keywords “emergence delirium”, “cognitive disorders”, “cognition”, “acute confusion”, “delirium”, “hallucination”, “dementia”, “mental”, “post-anaesthesia”, “symptom assessment”, “algorithm surveys and questionnaires”, “assess”, “audit”, “checklist”, “focus group”, “patient reported outcome measures”, “interview”, “instrument”, “QoL”, “screening”, “survey”, “pre-operative care”, “pre-procedure”, “post-operative”, “evaluation”, “validation”, “DSM” and “Diagnostic and Statistical Manual of Mental disorders”. The detailed search strategy is provided in Additional file 1.

### Study selection and data extraction

Potentially eligible studies were reviewed independently by two reviewers (EL and GH). Title review and abstracts were screened to determine if the inclusion/exclusion criteria were fulfilled. Any disagreements or queries were resolved by consulting the senior author (JW). Full text review of all included studies was conducted independently by the 3 reviewers (EL, GH and FA). Studies reporting the following were included: 1) Pre-operative biomarker testing for predicting POD, 2) imaging techniques to predict POD and 3) POD diagnosed by physician’s assessment or a validated tool. Exclusion criteria were paediatric studies, animal studies, non-English publications, intracranial or cardiac surgery, abstracts and studies that assessed emergence agitation as POD in their analysis or used non-validated tools for delirium assessment. We also excluded studies that examined post-operative biomarkers. Information extracted from each study were: first author and year of publication, sample size, mean age for POD and non-POD groups, country, type of surgery, elective or emergent, pre-operative dementia, pre-operative investigation for delirium, method of diagnosis for POD and day of POD diagnosis.

### Assessment of methodological quality

Each study was evaluated for risk of bias in 6 study domains using the Quality in Prognostic Studies (QUIPS) tool, (Additional file 2: Table S1) [6]. The QUIPS tool has been used widely for methodological quality in systematic reviews of risk prediction models. It assesses bias in prognostic factor studies; and rates 6 bias domains: 1) study participation (definition and description of the study subjects); 2) study attrition (study loss to follow-up); 3) prognostic factor measurement (quantitatively measured predictors of interest); 4) confounding measurement and handling; 5) outcome measurement (quantitatively measured outcome of interest) and; 6) statistical analysis (calibration and discrimination) as having high, moderate or low risk of bias [4]. Two reviewers (EL and GH) evaluated each study independently and discrepancies were resolved by analysing the weight of factors of bias described in the studies by a third author (FA).

## Results

The initial electronic database search retrieved 6214 potential studies with 3 additional studies retrieved from citations (Fig. 1). After removal of 2679 duplicates and reviewing the titles and abstracts, 34 studies were included for final full text review. Since the predictive tests and the manner of results reporting was heterogeneous [e.g. odds ratio (OR) vs. *P*-values only], and the number of studies for each specific test was low, we did not perform a quantitative analysis and limited our review to qualitative evaluation.

### Description of included studies and indices

POD assessment was performed using the Delirium Observation Screening Scale (DOSS) [7] Confusion Assessment Method (CAM) [8–36] or CAM-ICU [13, 24, 36], Delirium Rating Scale (DRS) [9, 15, 31, 37], Diagnostic and Statistical Manual of Mental Disorders (IV) [DSM IV] [7, 14, 36–39], or validated chart review [10, 21, 24, 34, 35]. Most studies assessed POD from the day of surgery, three studies assessed POD from post-operative day 2 onwards [8, 9, 15], and 6 studies did not specify the time of POD assessment [7, 23, 35–37, 40]. In studies which assessed delirium on the day of surgery, none mentioned whether they included PACU delirium or distinguished between emergence agitation or POD.

### Study characteristics

The main characteristics of the studies are shown in Table 1; serum biomarkers and Table 2; imaging tests. All were prospective cohort studies apart from one randomised controlled trial [41]. The 34 studies included data from 4424 patients. Nineteen studies described pre-operative serum biomarker tests [8, 10, 12–25, 37, 38, 41], 10 described CSF biomarker tests [7, 9, 11, 26–33] and 5 described imaging tests [34–36, 40, 41]. Four studies investigated several different biomarkers [9, 11, 14, 17].

**Table 1:** Summary of studies: Pre-operative serum tests

Author, Year, Country	Study design	n	Cut-off age	Type of surgery	Included patients with dementia or cognitive impairment	Multivariate regression analysis performed	Pre-operative tests	POD assessment, Days post-surgery assessment	POD- mean age, % female	No POD – mean age, % female	Conclusion	Pre-op Test Predictive of POD
Biomarkers												
Capri, 2014, Italy [8]	PC	74 (37 w/ POD, 37 w/o POD)	> 65	Any elective and emergency surgery, excluding cardiac	No	Yes	Plasma cytokine concentrations (TNF-a, IL-1b, IL-2, IL-6, IL-8, and IL-10)	CAM and DRS, D1,2,3 and 6	79.2 yr., 45.9%	76.4 yr., 54%	High serum IL-6 (> 9 pg/mL) independently predicts POD. OR 4.9, [1.6–14.63; *P* < 0.0005].	Yes
Westhoff, 2013, Netherlands [9]	PC	61	> 75	Emergency hip fracture	Yes	MA logistic regression analysis not performed but no significant correlation was found between age, cognitive function and levels of CSF cytokines.	42 CSF and serum cytokines and chemokines	CAM, DRS-R- 98, D1–5	82.9 yr., 69.9%	84.6 yr., 68.4%	Pre-op CSF Fms-like tyrosine kinase-3, IL-1r antagonist and IL-6. Pre-op serum IL-6 was significantly higher in patients with POD. Median levels in POD vs .no POD: 48.13 vs 23.16, *P* = 0.021.	Yes
Dillon, 2017, Israel and UK [10]	PC	566, Sub cohort from SAGE	> 70, Pooled cohort (combined match pairs)	Elective major non-cardiac	No	Conditional logistic regression and sensitivity analysis performed.	Serum CRP	CAM and validated chart review, POD = D1–2	77.6 yr., 56%	77.2 yr., 56%	Elevated pre-op CRP associated with POD (pre-op median paired difference with controlled subjects of 1.97 mg/L, P = 0.02).	Yes
Neerland, 2016, Norway & UK [11]	PC	151 Oslo 99, Edinburgh 52	> 60	Emergency hip fracture	Yes	No	Serum and CSF CRP, IL-6 and soluble IL-6 receptor	CAM O: D1–5, E: D1–4, 7, 10,14	85 yr., 70%	83 yr., 80%	Significantly high CSF levels of CRP in POD vs. no-POD, median 0.05mcg/mL vs. 0.01mcg/mL	Yes
Vasunilashorn, 2017, USA [12]	PC	560 (sub cohort from SAGE) POD = 25% of participants	> 70	Major non-cardiac surgery	No	No	Serum CRP	CAM, D1 until discharge	77.7 + − 5.0 yr., 60%	76.5 + − 5.2 yr., 58%	Subjects with pre-op CRP of ≥3 mg/L had a 1.5 greater risk of delirium than subjects with CRP; 4 more delirium days (*P* < 0.001) and 1.4 times greater risk of prolonged LOS.	Yes
Xiang, 2017, China [13]	PC	160 POD = 39(24.4%)	> 65	Laparoscopic surgery for colon carcinoma	No	Yes	Serum CRP	CAM-ICU D1–3 and 7	72.2 + − 5.8 yr., 41%	69.4 + − 7.1 yr., 39.7%	Pre-op CRP level: independent predictor of POD. POD vs. no POD CRP levels; 3.8 vs. 2.4 mg/L (OR: 5.87; 95% CI 2.22–11.4, *P* = 0.018).	Yes
Bohner, 2003, Germany [38]	PC	153	N/S	Elective Vascular surgery	No	No. Only Univariate analysis performed.	Pre-op: CRP, White cell count, platelets, LFTs, Creatinine, Urea, coagulation, Intra-op: BP, BG, glucose	DSM IV criteria, DRS > 12, D1–7	63.7 + − 10.3 yr.,	68.3 + − 8.5 yr.,	Pre-op CRP significantly higher in POD vs. no POD; 3.4 vs. 1.7 mg/dL, *P* = 0.03. AT III (98% vs. 106%, *p* = 0.02), lower Hb 13.7 vs. 14.3 g/dL, *P* = 0.02).	Yes
Lemstra, 2008, Netherlands [14]	PC	68	> 70	Elective hip replacement	Yes	No. Patients were matched for age, severe illness and MMSE score < 24 using a statistical analysis.	Serum CRP, IL-6, IGF-1	CAM and DSM-IV daily	80 yr., 55.6%	78.5 yr., 74%	No difference between POD vs. no POD levels: CRP = 5.3 vs. 3 mgL^− 1^, *P* = 0.523; IL-6 = 3.6 vs. 3.0 pgmL^− 1^, *P* = 0.121; IGF-1 = 14.4 vs. 12.9 nmolL ^− 1^, *P* = 0.675.	No
Shen, 2016, China [15]	PC	140	> 65	Elective open GI tumour resection	No	Yes	Serum IGF-1	CAM, DRS-R98. D2,3	73.8 yr., 52.8%	68.8 yr., 58.7%	Significantly low serum IGF-1 (POD vs. no-POD, 50.4 vs. 67 ng/mL), OR 2.52 (1.19–5.43).	Yes. Cut-off level 52.94 ng/ml, sensitivity of 80.8%, specificity of 80.6%.
Yen, 2016, USA [37]	PC	98	> 65	Elective knee replacement	No	Yes	Serum IGF-1	DSM-IV, DRS-R98	72.5 yr., 59%	73.7 yr., 50%	No association. Median levels in POD vs. no POD = 62.6 vs. 65.9 ng/mL, *P* = 0.141.	No
Cerejeira, 2011, Portugal [16]	PC	101	> 60	Elective total hip replacement	No	No	Plasma AChE and BuChE activity	CAM, D1,2,3	73.7 yr., 60%	72.7 yr., 46%	AChE and BuChE 24 and 32% lower in patients with vs. without POD, respectively. Pre-operative differences between the two groups were controlled.	Yes
Cerejeira, 2012, Portugal [17]	PC	101	> 60	Elective total hip replacement	No	No	Plasma AChE and BuChE and inflammatory mediator levels (CRP, IL-1b, and TNF-a)	CAM, D1,2,3	73.6 yr., 60%	72.7 yr., 45.3%	Low baseline plasma cholinesterase activity associated with POD and positively correlates with high CRP, IL-6 and Pro-inflammatory/ Anti-inflammatory ratio.	Yes
Chen, 2014, China [18]	PC	186	> 65	Emergency hip fracture	No	Yes	Plasma leptin	CAM, D1,2,3,7 and 1 month	80.1 yr., 72%	74.7 yr., 77%	Plasma leptin level is significantly lower in POD vs. no-POD, 4 vs. 7.5 ngmL^− 1^, *P* < 0.001). Hypoleptinaemia is an independent predictor of POD [OR 0.385, CI 0.28–0.517].	Yes. Sensitivity 72.2%, Specificity 91.7%
Cunningham, 2017, UK [19]	PC	315	> 65	Elective hip or knee replacement	No	Yes	Serum ApoE4 allele carriage and neuropsychological tests	CAM, D1,2,3	76.9 yr., 65%	74 yr., 27.1%	ApoE4 genotype is not associated with POD.	No
Leung, 2007, USA [20]	PC	190	> 65	Elective major non-cardiac	No	Yes	Serum apolipoprotein (APOE) genotype	CAM, D1–2	74.2 yr., NR	72.3 yr., NR	Presence of at least one copy of APOE e4 allele is associated with increased risk of POD that persists 2 days post-op. (OR 3.64, 95% CI 1.51–8.77).	Yes
Vasunilashorn, 2015, USA [21]	PC	557	> 70	Any Elective major non-cardiac	No	Sensitivity analyses performed to test if dementia has any influence on association between ApoE and POD.	Serum Apolipoprotein E: e2, e4 carrier vs non-carrier and three category ApoE genotypes (e3e3, e3e4, e4e4, e2e2, e3e2)	CAM and validated chart review daily			ApoE genotype has no association with incidence, severity or duration of POD, RR for E4 = 1.0, CI 0.7–1.	No
Torbergsen, 2015, Norway & Scotland [22]	PC	115	NR	Emergency hip fracture	No	Yes	Serum vitamin levels (Vitamin A, B1, B6, vitamin B12, Folic acid, vitamin C, D, K)	CAM, D1–5	84.8 yr., 71%	80.6 yr., 79%	Vitamin D deficiency (< 50 nmol/L) was independent predictor of POD. (Mean Vitamin D in POD vs. no-POD, 41 vs. 52 nmolL^−1^, P = 0.05).	Yes
Scholtens, 2017, Netherlands [23]	PC	144	> 65	Emergency hip fracture	No	No	Morning plasma melatonin	CAM	85.5 yr., 75%	82.5 yr., 69%	Morning melatonin not associated with POD (*p* = 0.35)	No
Scholtens, 2016, Netherlands [7]	PC	60	> 65	Emergency hip fracture	Yes	No but confounders (age and cognitive impairment) analysed and did not show any difference in CSF melatonin levels.	CSF melatonin	Delirium Observation Screening Scale, and DSM-IV, NS	86.4 yr., 83.3%	83.4 yr., 66%	Pre-operative CSF melatonin did not differ between POD and no-POD groups. No MA performed	No
Wyrobek, 2017, USA [24]	PC	77	> 70	Major Spine surgery Median age = 75 years, 47% were female	No	Yes	Intra-op serum brain derived neurotrophic factor (BDNF)	CAM, D1–4 CAM-ICU Validated chart review	NR	NR	Subjects with POD vs no. without POD had a greater percentage of BDNF decline from baseline, median 75% (IQR 51–82) vs. 50% (IQR 14–79, P = 0.03).	Yes
Zhang, 2017, China [25]	PC	700 111 (15.9%)	> 65	Elective non -cardiac surgery who were admitted to ICU	No	Yes	Pre-op serum albumin levels	CAM-ICU D1–7	76.2 + −7.8 yr., 47.7%	74.0 + − 6.6 yr., 38%	Pre-op severe hypoalbuminemia (< 30.0 g/L) was associated with increased risk of POD (OR 2.727, 95% CI 1.28–5.797, *P* = 0.009) .	Yes
Hall, 2016, Norway and Scotland [26]	PC	139 (Oslo- 85, Edinburgh- 54)	Oslo- > 60, Edin > 61	Emergency hip fracture	Yes	Yes	Serum and CSF neopterin.	CAM, D1–5	85 yr., 70%	82 yr., 77%	Higher pre-op CSF neopterin in POD vs. no-POD. (Median 29.6 vs 24.7 nmolL^− 1^, *P* = 0.003).	Yes
Hov, 2016, Norway [27]	PC	120	NR	Emergency hip fracture	Yes	No	Serum and CSF albumin. q-albumin (ratio of CSF albumin to serum albumin)	CAM, D1–5	85 yr., 70%	83 yr., 83%	CSF barrier dysfunction (q-albumin> 10.2). Significant difference in POD vs. no POD group, n = 11 (16% vs 0%, P = 0.022).	Yes
Watne, 2014, Norway [28]	PC	151 Oslo = 99 Edin = 52	Oslo- NR, Edin > 60, 84.	Emergency hip fracture	No	Yes	Serum and CSF anticholinergic activity	CAM D1–5	Oslo: 75% Edin: 60%	Oslo 72%, Edin: 75%	Serum or CSF anticholinergic activity is not an important mechanism in POD.	No
Watne, 2016, Norway [29]	PC	77	> 70	Emergency hip fracture	No	Yes	Serum and CSF monoamine precursors	CAM D1–5	86 yr., 70%	84 yr., 61%	Higher CSF monoamine precursors (tryptophan, tyrosine, phenylalanine, methionine, 5-HIAA) in delirium patients suggests high monoaminergic activity in CNS during delirium.	Yes
Hov 2017, Norway [30]	PC	Hip fracture = 98 Elective surgery = 50	> 70	Hip fracture and elective surgery (gynae, urology or orthopaedic	No	No	Serum and CSF S100B and phosphorylated tau (P-tau) concentration	CAM D1–5	Prevalent delirium- 85 yr., 75%, Incident delirium- 88 yr., 56%	84 yr., 78%	Significant difference in CSF S100B in patients with vs. without incident delirium (1.38 vs. 1.08 μgrams/L, P = 0.013).	Yes
Hall, 2013, Scotland [31]	PC	45	> 60	Emergency hip fracture	Yes	No	Serum and CSF S100B	CAM and DRS-R98, D1–4,7,10–14	81.3 yr., 63%	78.9 yr., 73%	No significant difference in log10 CSF S100B in POD vs no-POD during the 2-week period (mean: −0.239 vs −0.308 respectively; student’s t-test t = 1.25, df = 43, *P* = 0.218).	No
Westhoff, 2015, Netherlands [32]	PC	53	> 75, mean age 83.1, 67.9%	Emergency hip fracture	Yes	Yes	CSF proteins (low complement factor C3, fibulin-1, 1-beta-1,3 N-acetylglucosaminyl-transferase in POD, high neural cell adhesion molecule-2, fibrinogen, zinc-α-2-glycoproteinandhaptoglobin level in POD)	CAM, D1–5	NR	NR	Discrepencies of results between proteomic ccohort and validation cohort.	No
Witlox, 2011, Netherlands [33]	PC	76	> 75	Emergency hip fracture	Yes	Yes	CSF levels of senile plaques B amyloid and neurofibrillary tangles (Ptau)	CAM, D1–5	84.7 yr., 67%	82.4 yr., 67%	CSF markers for plaque and tangle formation are not strongly associated with POD.	No

*PC* Prospective cohort, *n* number, *POD* Post-operative delirium, *TNF* tumour necrosis factor, *IL* interleukin, *CAM* confusion assessment method, *DRS* delirium rating scale, *D1* day 1, *AChE* acetylcholinesterase, *BChE* butylcholinesterase, *CRP* c-reactive protein, *MMSE* mini mental state examination, *SAGE* Successful Ageing after Elective Surgery, *NR* not recorded, *AT III* angiotensin III, *Hb* hemoglobin, *5-HIAA* 5-hydroxyindoleacetic acid, *GI* gastrointestinal, *CSF* cerebral spinal fluid, *CAM-S* Confusion Assessment Method- Short Form, *DSM-IV* Diagnostic and Statistical Manual 4th, *IGF* Insulin Like Growth Factor, *PACU* post-anaesthesia care unit, *GA* general anaesthesia, *ICU* intensive care unit, *GABA* gamma amino butyric acid, *Fms* Fat Mobilising substance, *OR* odds ratio, *CBF* cerebral blood flow, *MA* multivariate analysis

**Table 2:** Summary of studies: imaging tests

Author, Year, Country	Study design	n	Cut-off age	Type of surgery	Included patients with dementia or cognitive impairment	Multivariate regression analysis performed	Pre-operative tests	POD assessment, Days post-surgery assessment	POD- mean age, % female	No POD – mean age, % female	Conclusion	Pre-op Test Predictive of POD
Imaging												
Cavallari, 2016, USA [34]	PC	136	> 70,	Elective non-cardiac	No	No but no significant difference were found between subjects (age, gender, vascular comorbidities)	MRI DTI of brain	CAM and validated chart review, daily	NR, 69%	NR, 56%	Significant association of DTI abnormalities in a variety of brain regions with POD. Mean differences in DTI indices between POD and non-POD = 3–8%, *P* < 0.05.	Yes
Hshieh, 2017, USA [35]	PC	146	> 70	Elective major non-cardiac	No	No	MRI of global and regional cerebral blood flow	CAM and validated chart review	NR, 69%	NR, 56%	No significant association between MRI of cerebral blood flow with POD in both adjusted and unadjusted analyses.	No
Miller, 2017, USA [40]	PC	142	> 65	Elective general surgery	No	Yes	CT of total psoas area	Retrospective medical record review	NR	NR	Low total psoas area is associated with POD (OR 3.12, 95% CI = 1.02–9.56, *P* = 0.046).	Yes
Root 2013, USA [41]	RC	48	NR	Anatomic lung resection for non-small cell carcinoma	No	No	MRI – WMHB and CA	Doctors, psychiatry and nurses detect confusion, disorientation, hyperactivity. D1–4	73.39 yrs., 56.5%	73.63 yrs., 54%	WMHB higher in POD vs no POD group (mean 0.01% vs 0.005% of total intracranial volume) *p* = 0.017.	Yes
Racine 2017, USA [36]	PC	145 (subset of SAGES)	> 70	Elective major non-cardiac	No	Yes	MRI of cortical thickness in Alzheimer’s Disease signature regions	CAM, validated chart review	77 +/− 4.3 yrs., 69%	76 +/− 4.6 yrs., 58%	Alzheimers’ Disease signature cortical thickness did not predict POD (OR = 1.15, 95% CI [0.8, 1.6].	No

*PC* prospective cohort, *RC* retrospective cohort, *n* number, *POD* post-operative delirium, *NR* not recorded, *CT* computed tomography, *OR* odds ratio, *CI* confidence interval, *p* probability, *RC* randomized controlled, *MRI* magnetic resonance imaging, *DTI* diffusion tensor imaging, *CAM* Confusion Assessment method, *D1–4* Day 1–4, *SAGES* Successful Ageing After Elective Surgery, *yrs.* years, *WMHB* white matter hyper-intensity burden, *CA* cerebral atrophy, *MA* Multivariate analysis

The studies were conducted in the United States, Europe and Asia. During data analysis, it was noted that several studies used the same patient population but examined different biomarkers. Specifically, 2 studies by Carejeira and colleagues [16, 17], and 6 studies used a subset from the Successful Aging after Elective Surgery (SAGE) study [10, 12, 21, 34–36]. The SAGE study was a prospective observational study evaluating the risk factors and long-term outcomes of delirium in 566 patients older than 70 years undergoing major non-cardiac surgery [39].

Five studies were major elective orthopaedic surgery [14, 16, 17, 19, 37], 14 emergency hip fracture surgery [7, 9, 11, 18, 22, 23, 26–29, 31–33], 10 major non-cardiac surgery [8, 10, 12, 20, 21, 25, 30, 34–36], 3 general surgery [13, 15, 40], 1 spine surgery [24], 1 vascular surgery [38] and 1 lung resection surgery [41]. All studies that examined CSF biomarkers involved emergency hip fracture patients. Most studies tested their biomarkers pre-operatively apart from 10 studies which tested their biomarkers in the pre-operative and post-operative period [10, 12, 13, 17].

### Risk of Bias

In general, risk of bias of all studies were low (Table 3). For the domain ‘study participation’, 6/34 (17.6%) had a moderate risk and 1 study had a high risk of bias. Confounding measurement was found to have moderate risk of bias in 23.5% (*n* = 8) of the studies. Five studies had moderate bias for attrition measurement and the main reasons were low numbers of eligible participants [18, 31–33]. Prognosis factor measurement and statistical analysis domains had low bias for all studies due to clear descriptions of valid prognostic factor measurements, and well-chosen statistical models that assessed the data in a non-selective manner respectively.

**Table 3:** Summary of risk of bias assessment

Domains	Low risk n (%)	Medium risk	High risk
Study participation	27 (79.4%)	6 (17.6%)	1 (2.9%)
Study attrition	29 (85.3%)	5 (14.7%)	0 (0)
Prognosis factor measurement	34 (100%)	0 (0)	0 (0)
Outcome measurement	33 (97.1%)	0 (0)	1 (2.9%)
Confounding measurement	26 (76.5%)	8 (23.5%)	0 (0)
Statistical analysis	34 (100%)	0 (0)	0 (0)

### Biomarkers

#### Inflammatory mediators

Ageing is associated with chronically low levels of inflammation [8]. The association of each biomarker with POD is summarized in Table 4.

**Table 4:** Summary of association of biomarkers and imaging tests with post-operative delirium

Biomarkers	Number of studies with positive association with post-operative delirium	References	Number of studies without an association with post-operative delirium	References
IL-6	2	6, 7	1	12
CRP	5	8, 9, 10, 11,,34	1	12
IGF-1	1	13	2	12,35
Angiontensin III	1	34		
Hemoglobin	1	34		
Reduced Cholinesterase activity	2	14, 15	0	
Brain derived neurotrophic factor (BDNF)	1	22	0	
Anticholinergic activity	0		1	26
Serum leptin	1	16		
APO E 4 genotype	1	18	2	17, 19
Hypovitaminsosis (Vitamin D deficiency)	1	20	0	
Melatonin	0		2	5, 21
Hypoalbuminemia	1	23	0	
Neopterin	1	24	0	
Monoamine precursor	1	27	0	
CSF Acute phase proteins	0		1	30
S100B	1	28	1	29
Plaques/ tangles	0		1	31
CSF barrier dysfunction	1	25	0	
Magnetic Resonance Imaging	2	32, 40	2	33, 41
Computed Tomography Imaging	1	38	0	

*IL-6* interleukin-6, *CRP* C-reactive protein, *IGF-1* insulin growth factor-1, *APO-E* alolipoprotein-E, *GABA* gamma aminobutyric acid

#### Il-6

Two studies showed elevated pre-operative serum IL-6 [8, 9], and 1 study did not find any association [14]. One study described their median IL-6 level in the POD group vs. non-POD group was 9 pgmL^− 1^ vs. 3.4 pgmL^− 1^ [8]. The other study showed no difference in CSF IL-6, however found significant difference in serum levels; (POD vs. non-POD: 48.1 vs. 23.1 pgmL^− 1^) [9]. The reason for the significant discrepancy in median absolute values between the two studies is not clear. However, the main difference between the two studies which may account for the different IL-6 levels was the use of pre-operative anti-inflammatory drugs; whereby the study which excluded its use reported higher IL-6 levels [9], while the study which reported lower IL-6 levels clearly indicated its main limitation was the absence of information on use of pre-operative anti-inflammatory drugs [8].

#### CRP

An association between raised CRP and POD was shown in 5 studies [10–13, 38], and 1 study [14] found no association. Vasinulashorn et al. [12] used a cut-off level for raised CRP as > 3 mgL^− 1^; where levels above this showed 1.5 times greater risk of developing delirium. Dillon et al. [10] showed higher CRP levels in POD; measured difference between POD and non-POD groups (median difference of 1.97 mgL^-1,^ *P* = 0.02). One study in general surgical patients showed higher levels in POD; (POD vs no POD, 3.8 mgL^− 1^ vs. 2.4 mgL^− 1^) [13], as well as another study in vascular patients (POD vs no POD; 3.4 vs. 1.7 mgL^− 1^) [38]. Neerland et al. [11] compared CSF CRP levels between the two groups; (POD vs. no POD, 0.05 vs. 0.01 mcgmL^-1)^. Based on these studies, a serum level cut-off of CRP > 3 mg/L is reasonable for predicting POD. The one study which found no association was small and may have been underpowered to show a difference [14]. This study also included patients with baseline cognitive impairment whereas the four studies excluded patients with cognitive impairment.

#### IGF-1

IGF-1 is a peptide growth hormone released mainly by the liver. It is neuroprotective; promoting neuronal survival and prevents oxidative stress by inhibiting cytokines. A reduced level may predispose the brain to cytotoxic effects of cytokines [15]. Low serum IGF-1 was significantly associated with POD in 1 study [15], however 2 studies [14, 37] found no association. Only 1 study evaluated the relationship between IGF-1 levels and severity of delirium, and did not find a significant association [37].

#### Neopterin

CSF neopterin is a marker of intrathecal immune activation and reflects CNS inflammation. It is synthesized upon stimulation of interferon gamma produced by activated T cells [42]. Increased neopterin levels were found in patients with delirium, even after adjustment for inflammatory state [43]. Significantly higher levels of pre-operative CSF neopterin were found in the POD group compared to non-POD group (29.6 vs 24.7 nmolL^− 1^, *P* = 0.03), even when participants with infection and malignant disease were excluded [26].

#### ApoE4 genotype

Apolipoprotein E (ApoE) is a component of plasma lipoprotein involved in central acetylcholine synthesis and maintenance of myelin and neuronal membranes during development or after injury. There are three isoforms of ApoE: E2, E3 and E4. The ApoE4 allele is most vulnerable to degradation compared to other isoforms, hence it carries the main genetic risk factor for late onset Alzheimer’s disease and is associated with greater risk of amyloid plaque deposition and cerebral amyloid angiopathy [44]. One study reported that the presence of one copy of the e4 allele increased risk of POD that persisted for 2 days post-operatively (28.3% vs. 11.1%; *P* = 0.005) [20], however two more recent studies reported no association [19, 21]. Interestingly, a recent systematic review of ApoE4 association with delirium concluded no significant association [45].

#### Anticholinergic activity

Cholinergic deficiency in the brain is known to play a role in delirium. Acetylcholine degradation not only causes cognitive dysfunction, but also induces inflammation in the central and peripheral nervous systems [46]. Impairment of its receptors might lead to cholinergic deficiency and delirium development [47]. One study investigated serum and CSF anticholinergic activity (AA) using muscarinic radio receptor bioassay, and expressed AA in terms of atropine equivalents (pmol/mL). It did not show an association with POD in the two centres in either serum AA in Oslo and in Edinburgh or CSF AA in Oslo and in Edinburgh [28].

#### Cholinesterases

Acetylcholinesterase and butylcholinesterases (AChE and BuChE) metabolise acetylcholine which results in a lack of cholinergic transmission; a proposed central mechanism of delirium [48]. Cerejeira et al. reported a significantly lower plasma AchE and BuChE of 24 and 32% respectively in the POD vs. non-POD group [16] and positive correlation between baseline plasma cholinesterase activity and pro-inflammatory/anti-inflammatory ratio [P/A, P/A = (IL 1B + TNF-A + IL-6 + IL-8)/ IL-10] with POD [17]. However, when assessing each mediator individually, no differences were observed between the 2 groups in the latter study [17]. The authors concluded that isolated levels of cytokines in the bloodstream do not necessarily capture the complex homeostatic dysfunction occurring during a delirium episode, and P/A ratios might be a more valuable index of cytokine response during a delirium episode [17].

#### Blood brain barrier dysfunction

Blood brain barrier dysfunction has been described in dementia [49], but not many studies have described its role in delirium. Pre-operative CSF and serum albumin ratio as a marker of blood brain barrier (BBB) dysfunction (q albumin = ratio of CSF albumin to serum albumin, q > 10 = CSF barrier dysfunction) has been examined in one study. A significant difference was found in q albumin between the POD and the non-POD group after hip fracture surgery [*n* = 11 (16% vs. 0%), *P* = 0.022] [27].

#### Melatonin

Melatonin is responsible for regulation of circadian rhythm and sleep-wake cycle; the latter being a core feature in delirium. It also inhibits the aggregation of the amyloid beta protein into neurotoxic micro-aggregates responsible for the neurofibrillary tangles in Alzheimer’s disease and prevents hyperphosphorylation of the tau protein [50]. Two studies however failed to show an association between pre-operative melatonin levels and POD [10, 23].

#### Monoamine precursors

Monoamines such as dopamine, noradrenaline and serotonin have important roles in attention and cognition [51]. The rate of monoamine synthesis in the brain is determined by monoamine precursors (tryptophan, tyrosine and phenylalanine). One study examined the association between POD and amino acids and/or monoamine metabolites (5-hydroxyindoleacetic acid; 5-HIAA) levels in the CSF in patients with and without pre-existing dementia. Significantly higher CSF levels of tryptophan, 0.8 vs. 1.1 μmolL^− 1^, *P* = 0.042; tyrosine, 5 vs. 8 μ molL^− 1^, *P* = 0.028; phenylalanine, 9 vs. 14 μ molL^− 1^, *P* = 0.014; methionine, 3 vs. 4 μmolL^− 1^, *P* = 0.03 and monoamine metabolite 5-HIAA, 138 vs. 170 nmolL^− 1^, *P* = 0.048 were found in the POD vs. the non-POD group after hip fracture surgery [29]. These findings were found in patients without pre-existing dementia. When patients with and without dementia were analysed together, only CSF methionine and serum taurine were significantly different between the POD and non-POD group. The study suggested that increased monoaminergic activity is associated with delirium in hip fracture patients.

#### CSF protein

Acute phase proteins (complement c3, fibrinogen and haptoglobin) may be associated with the inflammatory process in delirium [52]. Complement factor C3, contactin-1, fibulin-1 and I-beta-1,3-N-acetylglucosaminyl transferase were significantly lower, while neural cell adhesion molecule-2, fibrinogen, zinc-α-2-glycoprotein and haptoglobin levels were significantly higher in the POD vs. control group in their proteomic cohort after hip fracture surgery, but failed to demonstrate this in their validation cohort [32]. The authors concluded that their inconsistent results were due to minor differences between the two cohorts (patients in the validation group were older than the proteomic group) and also due the complexity of the pathophysiology of delirium.

#### CSF S100B

S100B is a calcium binding protein in the CNS mainly secreted by astrocytes under metabolic stress. Elevated levels have been implicated as a marker of acute brain damage [53]. One study in both hip fracture surgery and the elective surgery showed a significant difference (POD vs. no POD, 1.38 vs. 1.08 μgramL^− 1^, *P* = 0.013) [30]. However, this difference was confined to patients with pathological concentrations of phosphorylated-tau (> 60 ng/L) - an abnormally phosphorylated protein; its presence in the CSF reflects early signs of Alzheimer’s dementia [54]. Another study however found no significant difference in S100B concentration between POD and non-POD groups having hip fracture surgery [31]. The reason for inconsistencies were not clear, but the populations in both studies differed; with a higher proportion of nursing home and dementia patients in the positive study [30].

#### Neurofibrillary plaques

Senile plaques composed of beta-amyloid (AB1–42) and neurofibrillary tangles (phosphorylated tau) are commonly associated with the pathological process in Alzheimer’s dementia [51]. The relationship of Alzheimer’s disease and susceptibility of POD is not well understood, however, in one study, CSF markers for plaques and tangles were not significantly associated with POD risk in elderly hip fracture patients [33].

#### Leptin

Leptin modulates immune response, and hypoleptinaemia can increase the production of pro-inflammatory cytokines that could result in cognitive impairment and delirium [55]. Low plasma leptin was associated with POD after femoral neck fracture surgery in one study (POD vs. no POD: 4.6 vs 7.5 ngmL^− 1^, *p* < 0.001) [18].

#### Hypovitaminosis

Vitamin D is a steroid hormone, which acts on neuroanatomic areas that sub-serve cognition. Hypovitaminosis and its link with cognitive impairment has been described in a few studies [56, 57]. Theoretically, Vitamin D may have a role in protecting the brain from delirium by preventing oxidative stress in hippocampal neurons [58] and promotes neuroprotection by modulating the production of choline acetyltransferase [59]. One study examining serum vitamin levels in hip fracture patients showed that Vitamin D deficiency (< 50 nmolL^− 1^) was an independent predictor of POD; mean Vitamin D levels in POD vs. no POD: 41 vs. 52 nmolL^− 1^, *p* = 0.05 [22].

#### Hypoalbuminemia

Hypoalbuminemia is used as a marker of malnutrition and several studies have shown hypoalbuminemia as a risk factor for POD [60, 61]. Severe hypoalbuminaemia (< 30 g/L) but not mild (30.1 - 35 g/L) or moderate (35.1 - 40 g/L) hypoalbuminemia was significantly associated with a two-fold higher risk of POD [25].

#### Brain derived neurotrophic factor (BDNF)

BDNF is a protein that influences neuroplasticity and neurotransmission, learning, memory and cognition. One study showed that greater decline in BDNF levels from baseline was associated with an increased incidence of delirium in patients undergoing spine surgery; POD vs no POD 75% vs 50%, *p* = 0.03 [24].

#### Angiotensin III (AT III) and haemoglobin

One study found that a lower AT III level (POD vs no POD, 98% vs. 106%, *p* = 0.02); and haemoglobin level pre-operatively (POD vs no POD, 13.7 vs. 14.3 g/dL, *P* = 0.02) increase the risk of developing POD in vascular surgery [38].

#### Imaging

Integration of imaging results with information such as structural integrity or changes, vascular profile, or neurotransmitter imbalances can give rise to computational modelling of POD.

#### Magnetic resonance imaging (MRI)

Various techniques in MRI have been reported in predicting POD. Diffusion tensor imaging is a MRI technique that shows microstructural integrity of the brain parenchyma and has been applied to map underlying functional impairments. A significant association of premorbid diffusion tensor imaging abnormalities with delirium incidence and severity in a variety of brain regions, particularly the cerebellum was shown in one study [34]. In a cohort of patients undergoing lung resection surgery, cerebral MRI showed higher proportion of white matter hyper-intensities in the POD vs. non-POD group, (mean 0.01% vs 0.005% of total intracranial volume, *P* = 0.017) [41]. However, two studies involving MRI; one study on global and regional cerebral blood flow [35], and another study of cortical thickness in Alzheimer’s disease signature region [36] showed no association with POD.

#### Computed tomography imaging (CT)

One study found that low total psoas area (TPA), a marker of surgical frailty was associated with an almost 3-fold increased risk of POD [40].

The current evidence on pre-operative biomarkers associated with POD are summarised in Table 4.

## Discussion

Biomarkers can be useful to improve understanding of pathophysiology of POD, develop new treatment for POD, predict possible long term effects of delirium, stratify high risk patients and allow informed decision making for patients about their risks for surgery. Many biomarkers associated with dementia or hypothesized causes of post-operative delirium have been investigated.

In this review, CRP was the most promising biomarker for predicting POD. Although there is variability in CRP measurement and cut-off levels in each study, the slightly raised CRP levels that were predictive in the studies suggests that there may be an association of heightened innate inflammatory response leading to POD. There is growing evidence of raised innate inflammation associated with POD as evidenced by elevation of pre-operative inflammatory markers such as CRP, IL-6 and neopterin. One hypothesis suggests an exaggerated activation of the innate immune system results in functional changes in neurons which may lead to cognitive and behavioural changes [62]. The degree of neuro-inflammation tends to increase with age, which may explain why elderly individuals are more prone to developing POD when administered anaesthesia [63]. Predisposed individuals who experience stressors such as during infection or surgery may also exhibit crossing of systemic inflammatory mediators across the blood-brain barrier and activation of brain microglia, which eventually leads to neuro-inflammation and delirium [12, 64].

CRP is a non-specific acute phase marker in inflammation, infection and tissue damage. Although raised CRP may be non-specific to POD, the initial findings are encouraging for further research. Other biomarkers that were found to be associated with POD include BDNF, reduced cholinesterase activity, serum leptin, hypoalbuminaemia and vitamin D deficiency. However, we cannot draw any conclusions as to their roles in POD due to the limited number of studies.

The results for CSF biomarkers and imaging as predictors for POD are inconclusive. The studies that examined CSF biomarkers were for patients undergoing spinal anaesthesia, thus the patients did not require an additional lumbar puncture to obtain CSF. The risks and invasiveness of obtaining CSF (if a patient was not already undergoing spinal anaesthesia) and costs applied to imaging may limit their practicality in clinical practice.

### Strengths and limitations

The strengths of this review include the comprehensive literature search using different search terms from several large databases and the total number of patients included from geographically diverse areas.

The limitations of the review are that most studies were relatively small and some biomarkers were only studied in one study, hence limiting quantitative assessment and meta-analysis of results. Some studies used the same cohort of patients to test different biomarkers. Secondly, we restricted our search only to English language articles. Most studies were conducted in orthopaedic surgery; therefore, the results may not be generalised to other types of surgery. Another limitation is that some studies included patients with pre-existing cognitive impairment or dementia [7, 9, 11, 14, 26, 27, 31–33]. However, no statistical difference were found in pre-operative cognitive scores between control and POD groups in all the studies. Some studies did not perform multivariate analysis which may not account fully for potential confounders [7, 9, 11, 12, 14, 16, 17, 21, 23, 27, 30–32, 34, 41]. The timing of POD assessment also varied, and no study mentioned if they included PACU delirium. However, whether delirium in PACU is considered POD is subject to debate, and although its assessment is different (CAM-ICU has a higher specificity than sensitivity for delirium when used in PACU) [2], the relatively high incidence of PACU delirium (16.4%) [3] may be relevant in accounting for the accurate incidence of POD.

Therefore, further research should incorporate a variety of surgical procedures, align timing of POD assessment including PACU delirium, using multivariate models and adjust for confounding factors.

## Conclusions

The current literature regarding pre-operative biochemical markers and imaging tests for predicting post-operative delirium is still in its infancy. There is some evidence that serum CRP levels > 3 mgL^− 1^ may predict a high risk of developing POD, and incorporating it along with other patient and surgical predisposing factors of POD may increase the identification of high risk patients. However, this should be interpreted with caution due to heterogeneity of predictive test values and the small sample size of the studies precluded sensitivity analysis to demonstrate significant association. On the other hand, delineating specific biomarkers may also lead to forming composite markers (combining 2 or more biomarkers) which could increase predictive value. From a clinical perspective, utilising predictive biomarkers may allow better clinical decision making and management of high risk patients to specific recovery units.

## Additional files

Additional file 1: Search strategy. (DOCX 129 kb)

Additional file 2: Supplementary Table S1. QUIPS Risk of bias assessment.. (DOCX 88 kb)

## Abbreviations

QoL: Quality of life
CAM-ICU: Confusion assessment methd-intensive care unit
5-HIAA: 5-hydroxyindoleacetic acid
6 RCT: Randomised control trial
AB1-42: Amyloid-b 1-42
AChE: Acetylcholinesterase
ApoE: Alolipoprotein-E
AT III: Angiotensin-III
BBB: Blood brain barrier
BDNF: Brain derived neurotrophic factor
BuChE: Butylcholinesterase
CAM: Confusion Assessment Method
CRP: C-Reactive protein
CSF: Cerebral spinal fluid
CT: Computed tomography
DRS: Delirium rating scale
DSM: Diagnostic and statistical manual for mental disorders
IGF-1: Insulin-like growth factor-1
IL-10: Interleukin-10
IL-1B: Interleukin-1B
IL-6: Interleukin-6
IL-8: Interleukin-8
MRI: Magnetic resonance 13 imaging
OR: Odds ratio
P/A: Pro-inflammatory/anti-inflammatory ratio
POD: Post-operative delirium
PRISMA: Preffered reporting items for systematic review
QUIPS: Quality in Prognostic Studies
SAGE: Successful ageing after elective surgery
TNF-A: Tumour necrosis factor-Alpha
TPA: Total psoas area
