# Supplementary material for: Pre-operative biomarkers and imaging tests as predictors of post-operative delirium in non-cardiac surgical patients: a systematic review
Source: BMC Anesthesiol. 2019 Feb 23;19:25. doi: 10.1186/s12871-019-0693-y (PMC6387490; doi:10.1186/s12871-019-0693-y)
Supplement: Supplementary file 1 — Search strategy. (DOCX 129 kb) [file 12871_2019_693_MOESM1_ESM.docx]

Search strategy saved as: 2018-06-22 JW -REVISED 2- Preop Assessment and Postoperative Delirium and Studies with Biomarkers - FINAL Searches

# Medline

Ovid MEDLINE(R) 1946 to June Week 3 2018

| **#** | **Searches** | **Results** |
| --- | --- | --- |
| 1 | (postoperative adj1 delirium).mp,kw. | 831 |
| 2 | (post-operative adj1 delirium).mp,kw. | 79 |
| 3 | Emergence Delirium/ [ New MeSH as of 2017 ] | 61 |
| 4 | cognition disorders/ or mild cognitive impairment/ | 69243 |
| 5 | (cognitiv* adj2 impair*).mp,kw. | 48205 |
| 6 | (disorder* adj2 cognit*).mp,kw. | 64142 |
| 7 | (cognitiv* adj2 status).mp,kw. | 4413 |
| 8 | (cognitiv* adj2 dysfunc*).mp,kw. | 18745 |
| 9 | (cognitiv* adj2 function*).mp,kw. | 47056 |
| 10 | dyscognitiv*.mp,kw. | 40 |
| 11 | cognitive*.mp,kw. | 259374 |
| 12 | exp Cognition/ | 140010 |
| 13 | cognition*.mp,kw. | 164252 |
| 14 | Confusion/ | 4501 |
| 15 | (confuse? or confusing or confusion*).mp,kw. | 46920 |
| 16 | Delirium/ | 7776 |
| 17 | delirium?.mp,kw. | 13259 |
| 18 | delirius.mp,kw. | 1 |
| 19 | delirious*.mp,kw. | 1087 |
| 20 | Hallucinations/ | 10176 |
| 21 | hallucinat*.mp,kw. | 15698 |
| 22 | exp Dementia/ | 146632 |
| 23 | dementia?.mp,kw. | 96031 |
| 24 | demented??.mp,kw. | 7542 |
| 25 | dementat*.mp,kw. | 3 |
| 26 | demenc???.mp,kw. | 1393 |
| 27 | (intellectual* adj2 declin*).mp,kw. | 289 |
| 28 | amentia???.mp,kw. | 87 |
| 29 | (mental* adj2 deteriorat*).mp,kw. | 1373 |
| 30 | (mental* adj2 acuit*).mp,kw. | 83 |
| 31 | (post-an?esth* adj1 excit*).mp,kw. | 1 |
| 32 | (postan?esth* adj1 excit*).mp,kw. | 12 |
| 33 | (emergence adj1 agitat*).mp,kw. | 243 |
| 34 | (emergence adj1 excit*).mp,kw. | 9 |
| 35 | (acute?? adj1 confusion?? adj1 state?).mp,kw. [ added June 20 2018 ] | 397 |
| 36 | (toxic-metabolic adj1 encephalopath*).mp,kw. [ added June 20 2018 ] | 46 |
| 37 | (acute adj1 brain adj1 syndrome?).mp,kw. [ added June 20 2018 ] | 50 |
| 38 | (acute adj1 brain adj1 fail*).mp,kw. [ added June 20 2018 ] | 15 |
| 39 | (acute adj1 organ?? adj1 psychos#s).mp,kw. [ added June 20 2018 ] | 25 |
| 40 | (acute adj1 organ?? adj1 brain syndrome?).mp,kw. [ added June 20 2018 ] | 37 |
| 41 | (ICU adj2 psychos#s).mp,kw. [ added June 20 2018 ] | 26 |
| 42 | (Intensive Care adj3 psychos#s).mp,kw. [ added June 20 2018 ] | 20 |
| 43 | (metabolic adj1 encephalopath*).mp,kw. [ added June 20 2018 ] | 543 |
| 44 | pseudosenilit*.mp,kw. [ added June 20 2018 ] | 1 |
| 45 | pseudo-senilit*.mp,kw. [ added June 20 2018 ] | 3 |
| 46 | (reversab* adj2 dementia?).mp,kw. [ added June 20 2018 ] | 1 |
| 47 | (toxic adj2 psychos#s).mp,kw. [ added June 20 2018 ] | 640 |
| 48 | or/1-47 [ ~~ Cognition / Cognitive Impairment / Confusion / Delirium ~~ ] | 554069 |
| 49 | Symptom Assessment/ | 2958 |
| 50 | Algorithms/ | 224838 |
| 51 | Data Collection/ | 86825 |
| 52 | Focus Groups/ | 24615 |
| 53 | Health Care Surveys/ | 29620 |
| 54 | Health Impact Assessment/ | 473 |
| 55 | Health Status Indicators/ | 22386 |
| 56 | Health Surveys/ | 57011 |
| 57 | Interviews As Topic/ | 54300 |
| 58 | Narration/ | 7132 |
| 59 | Patient Acuity/ | 766 |
| 60 | Patient Reported Outcome Measures/ | 1631 |
| 61 | "Quality of Life"/ | 162493 |
| 62 | "Severity Of Illness Index"/ | 215454 |
| 63 | Sickness Impact Profile/ | 6912 |
| 64 | "Surveys and Questionnaires"/ | 399335 |
| 65 | algorithm*.mp,kw. | 286751 |
| 66 | assess*.mp,kw. | 2468986 |
| 67 | audit?.mp,kw. | 41680 |
| 68 | checklist?.mp,kw. | 27296 |
| 69 | check-list?.mp,kw. | 2494 |
| 70 | evaluat*.mp,kw. | 2958572 |
| 71 | exam*.mp,kw. | 2599073 |
| 72 | focus group*.mp,kw. | 35195 |
| 73 | (health adj2 determinant*).mp. | 6493 |
| 74 | HRQoL.mp,kw. | 10989 |
| 75 | index*.mp,kw. | 841438 |
| 76 | indices.mp,kw. | 131092 |
| 77 | indicator?.mp. | 290021 |
| 78 | interview*.mp,kw. | 298103 |
| 79 | instrument?.mp,kw. | 165126 |
| 80 | inventories.mp,kw. | 5042 |
| 81 | inventory.mp,kw. | 78715 |
| 82 | measur*.mp,kw. | 2766146 |
| 83 | metric?.mp,kw. | 33084 |
| 84 | narrati*.mp,kw. | 30323 |
| 85 | nomogram*3.mp,kw. | 6351 |
| 86 | patient analys#s.mp,kw. | 639 |
| 87 | poll?.mp,kw. | 2666 |
| 88 | QoL.mp,kw. | 26108 |
| 89 | quality of life.mp,kw. | 249201 |
| 90 | questionnaire?.mp,kw. | 570653 |
| 91 | rating?.mp,kw. | 176701 |
| 92 | (scale or scales).mp,kw. | 587233 |
| 93 | screening?.mp,kw. | 465801 |
| 94 | (score or scores or scored or scoring?).mp,kw. | 689786 |
| 95 | status exam*.mp,kw. | 1553 |
| 96 | survey*.mp,kw. | 804963 |
| 97 | (test or tests).mp,kw. | 2241343 |
| 98 | (tool? or toolkit? or tool-kit?).mp,kw. | 489811 |
| 99 | psychological tests/ or trail making test/ | 36608 |
| 100 | exp Psychiatric Status Rating Scales/ | 78549 |
| 101 | Geriatric Assessment/ | 23874 |
| 102 | Mass Screening/ | 93983 |
| 103 | (battery or batteries).mp,kw. | 39425 |
| 104 | "diagnostic and statistical manual of mental disorders".mp,kw,kf. | 20049 |
| 105 | "diagnostic & statistical manual of mental disorders".mp,kw,kf. | 29 |
| 106 | DSM.mp,kw,kf. | 44262 |
| 107 | (DSM1 or DSM2 or DSM3 or DSM4 or DSM5).mp,kw,kf. | 64 |
| 108 | or/49-107 [ Assessment or Measurement or Scales or Indexes & related terms; QoL & HRQoL added ] | 10256603 |
| 109 | 48 and 108 [ Delirium + Assessment ] | 361681 |
| 110 | Preoperative Care/ | 57820 |
| 111 | preoperative period/ | 5198 |
| 112 | (preoperat* or pre-operat*).mp,kw. | 275968 |
| 113 | (before adj2 surg*).mp,kw. | 41209 |
| 114 | (before adj2 procedur*).mp,kw. | 5784 |
| 115 | (before adj2 operat????).mp,kw. | 14240 |
| 116 | (prior adj2 procedur*).mp,kw. | 1612 |
| 117 | (prior adj2 operat????).mp,kw. | 2098 |
| 118 | (prior adj1 surg*).mp,kw. | 2842 |
| 119 | pre-procedur*.mp,kw. | 1465 |
| 120 | preprocedur*.mp,kw. | 2589 |
| 121 | preintervention*.mp,kw. | 2747 |
| 122 | pre-intervention*.mp,kw. | 2769 |
| 123 | pre-surg*.mp,kw. | 2747 |
| 124 | "pre- and post-surgery".mp,kw. | 362 |
| 125 | "pre- and post-surgical".mp,kw. | 298 |
| 126 | "pre- and postsurgery".mp,kw. | 194 |
| 127 | "pre- and postsurgical".mp,kw. | 308 |
| 128 | or/110-127 [ Preoperative & related terms ] | 333314 |
| 129 | 109 and 128 [ Delirium + Assessment + Preoperative ] | 4449 |
| 130 | exp Surgical Procedures, Operative/ | 2865838 |
| 131 | su.fs. ["surgery" floating subheading ] | 1830798 |
| 132 | exp postoperative care/ | 56605 |
| 133 | exp Postoperative Period/ | 48856 |
| 134 | exp Postoperative Complications/ | 494989 |
| 135 | "after surgery".mp,kw. | 117844 |
| 136 | "after surgical*".mp,kw. | 23823 |
| 137 | (following adj2 (surgery or surgeries or surgical)).mp,kw. | 39192 |
| 138 | (postoperat* or post-operat*).mp,kw. | 736908 |
| 139 | post-intervention*.mp,kw. | 8652 |
| 140 | postintervention*.mp,kw. | 6002 |
| 141 | Post-procedur*.mp,kw. | 4137 |
| 142 | postprocedur*.mp,kw. | 5262 |
| 143 | post-surg*.mp,kw. | 11657 |
| 144 | postsurg*.mp,kw. | 14837 |
| 145 | or/130-144 [ Postoperative Care/Period/Complications ] | 3613420 |
| 146 | 129 and 145 [ Delirium + Assessment + Preoperative + Postoperative ] | 3946 |
| 147 | Randomized controlled trial.pt. | 461808 |
| 148 | exp Randomized controlled trial/ | 462129 |
| 149 | exp Randomized Controlled Trials as Topic/ | 118687 |
| 150 | Pragmatic Clinical Trial.pt. | 777 |
| 151 | Pragmatic Clinical Trial/ | 777 |
| 152 | Pragmatic Clinical Trials As Topic/ | 190 |
| 153 | (pragmatic adj2 (trial? or study or studies)).mp,kw. | 2222 |
| 154 | random*.mp,kw. | 1057386 |
| 155 | Double-Blind Method/ | 146082 |
| 156 | ((single or double or triple or treble) adj3 (blind* or mask*)).mp,kw. | 202979 |
| 157 | doubleblind*.mp,kw. | 198 |
| 158 | Placebos/ | 33968 |
| 159 | Placebo*.mp,kw. | 192441 |
| 160 | cohort*.mp,kw. | 481887 |
| 161 | evaluation studies.pt. | 235121 |
| 162 | (evaluation adj1 (study or studies)).mp,kw. | 360202 |
| 163 | validation studies.pt. | 89601 |
| 164 | (validation adj1 (study or studies)).mp,kw. | 98360 |
| 165 | clinical trial.pt. | 510282 |
| 166 | Controlled Clinical Trial.pt. | 92422 |
| 167 | Controlled Clinical Trial/ | 92422 |
| 168 | Controlled Clinical Trials As Topic/ | 5337 |
| 169 | (controlled adj1 clinical adj2 (trial? or study or studies)).mp,kw. | 119922 |
| 170 | meta-analysis.pt. | 89071 |
| 171 | meta-analysis/ | 89071 |
| 172 | meta-analysis as topic/ | 16244 |
| 173 | (meta-anal* or metanal* or metaanal*).mp,kw. | 132096 |
| 174 | (systematic adj4 (review or reviews or overview or overviews)).mp,kw. | 100556 |
| 175 | (overview? adj4 (review or reviews)).mp,kw. | 11358 |
| 176 | exp Cohort Studies/ | 1748783 |
| 177 | exp Case-Control Studies/ | 920578 |
| 178 | (case control* adj2 (study or studies)).mp,kw. | 269457 |
| 179 | Longitudinal Studies/ | 115743 |
| 180 | (longitudinal* adj2 (study or studies)).mp,kw. | 144451 |
| 181 | Prospective Studies/ | 474077 |
| 182 | (prospective* adj2 (study or studies)).mp,kw. | 547755 |
| 183 | Cross-Sectional Studies/ | 267155 |
| 184 | (cross-sectional* adj2 (study or studies)).mp,kw. | 285766 |
| 185 | case series.mp,kw. | 48672 |
| 186 | Retrospective Studies/ [added May 9 ] | 690304 |
| 187 | (retrospective* adj2 (study or studies)).mp,kw. [ added May 9 ] | 717205 |
| 188 | "sensitivity and specificity"/ or "predictive value of tests"/ or roc curve/ [ added May 9 ] | 502494 |
| 189 | or/147-188 [ HEDGE - Therapy Question with All Types of Studies ] | 4135824 |
| 190 | 146 and 189 [ Delirium + Assessment + Preoperative + Postoperative + Studies ] | 2547 |
| 191 | exp Biomarkers/ | 651208 |
| 192 | biomarker?.mp. | 420298 |
| 193 | bio-marker?.mp. | 430 |
| 194 | (marker or markers).mp. | 613911 |
| 195 | exp proteomics/ or proteogenomics/ | 43263 |
| 196 | proteomic??.mp. | 67067 |
| 197 | proteogenomic??.mp. | 410 |
| 198 | exp Endophenotypes/ | 812 |
| 199 | endophenotyp???.mp. | 3429 |
| 200 | exp Genetic Markers/ | 52676 |
| 201 | or/191-200 [ Biomarkers & related terms ] | 1159380 |
| 202 | 48 and 128 and 145 and 189 and 201 [ Delirium + Assessment + Preoperative + Postoperative + Studies + Biomarkers ] | 136 |
| 203 | limit 202 to english language | 133 |
| 204 | remove duplicates from 203 | 133 |

# Medline In-Process

Ovid MEDLINE(R) Epub Ahead of Print and In-Process & Other Non-Indexed Citations June 21, 2018

| **#** | **Searches** | **Results** |
| --- | --- | --- |
| 1 | (postoperative adj1 delirium).mp,kw. | 250 |
| 2 | (post-operative adj1 delirium).mp,kw. | 35 |
| 3 | Emergence Delirium/ [ New MeSH as of 2017 ] | 0 |
| 4 | cognition disorders/ or mild cognitive impairment/ | 0 |
| 5 | (cognitiv* adj2 impair*).mp,kw. | 9915 |
| 6 | (disorder* adj2 cognit*).mp,kw. | 1087 |
| 7 | (cognitiv* adj2 status).mp,kw. | 708 |
| 8 | (cognitiv* adj2 dysfunc*).mp,kw. | 2253 |
| 9 | (cognitiv* adj2 function*).mp,kw. | 9037 |
| 10 | dyscognitiv*.mp,kw. | 10 |
| 11 | cognitive*.mp,kw. | 49900 |
| 12 | exp Cognition/ | 0 |
| 13 | cognition*.mp,kw. | 14120 |
| 14 | Confusion/ | 0 |
| 15 | (confuse? or confusing or confusion*).mp,kw. | 6730 |
| 16 | Delirium/ | 0 |
| 17 | delirium?.mp,kw. | 2008 |
| 18 | delirius.mp,kw. | 0 |
| 19 | delirious*.mp,kw. | 143 |
| 20 | Hallucinations/ | 0 |
| 21 | hallucinat*.mp,kw. | 1550 |
| 22 | exp Dementia/ | 0 |
| 23 | dementia?.mp,kw. | 13433 |
| 24 | demented??.mp,kw. | 577 |
| 25 | dementat*.mp,kw. | 1 |
| 26 | demenc???.mp,kw. | 306 |
| 27 | (intellectual* adj2 declin*).mp,kw. | 17 |
| 28 | amentia???.mp,kw. | 15 |
| 29 | (mental* adj2 deteriorat*).mp,kw. | 127 |
| 30 | (mental* adj2 acuit*).mp,kw. | 9 |
| 31 | (post-an?esth* adj1 excit*).mp,kw. | 0 |
| 32 | (postan?esth* adj1 excit*).mp,kw. | 0 |
| 33 | (emergence adj1 agitat*).mp,kw. | 80 |
| 34 | (emergence adj1 excit*).mp,kw. | 1 |
| 35 | (acute?? adj1 confusion?? adj1 state?).mp,kw. [ added June 20 2018 ] | 60 |
| 36 | (toxic-metabolic adj1 encephalopath*).mp,kw. [ added June 20 2018 ] | 6 |
| 37 | (acute adj1 brain adj1 syndrome?).mp,kw. [ added June 20 2018 ] | 1 |
| 38 | (acute adj1 brain adj1 fail*).mp,kw. [ added June 20 2018 ] | 2 |
| 39 | (acute adj1 organ?? adj1 psychos#s).mp,kw. [ added June 20 2018 ] | 1 |
| 40 | (acute adj1 organ?? adj1 brain syndrome?).mp,kw. [ added June 20 2018 ] | 2 |
| 41 | (ICU adj2 psychos#s).mp,kw. [ added June 20 2018 ] | 0 |
| 42 | (Intensive Care adj3 psychos#s).mp,kw. [ added June 20 2018 ] | 4 |
| 43 | (metabolic adj1 encephalopath*).mp,kw. [ added June 20 2018 ] | 46 |
| 44 | pseudosenilit*.mp,kw. [ added June 20 2018 ] | 0 |
| 45 | pseudo-senilit*.mp,kw. [ added June 20 2018 ] | 0 |
| 46 | (reversab* adj2 dementia?).mp,kw. [ added June 20 2018 ] | 0 |
| 47 | (toxic adj2 psychos#s).mp,kw. [ added June 20 2018 ] | 7 |
| 48 | or/1-47 [ ~~ Cognition / Cognitive Impairment / Confusion / Delirium ~~ ] | 72395 |
| 49 | Symptom Assessment/ | 0 |
| 50 | Algorithms/ | 0 |
| 51 | Data Collection/ | 0 |
| 52 | Focus Groups/ | 0 |
| 53 | Health Care Surveys/ | 0 |
| 54 | Health Impact Assessment/ | 0 |
| 55 | Health Status Indicators/ | 0 |
| 56 | Health Surveys/ | 0 |
| 57 | Interviews As Topic/ | 0 |
| 58 | Narration/ | 0 |
| 59 | Patient Acuity/ | 0 |
| 60 | Patient Reported Outcome Measures/ | 0 |
| 61 | "Quality of Life"/ | 0 |
| 62 | "Severity Of Illness Index"/ | 0 |
| 63 | Sickness Impact Profile/ | 0 |
| 64 | "Surveys and Questionnaires"/ | 0 |
| 65 | algorithm*.mp,kw. | 52054 |
| 66 | assess*.mp,kw. | 330392 |
| 67 | audit?.mp,kw. | 4421 |
| 68 | checklist?.mp,kw. | 5393 |
| 69 | check-list?.mp,kw. | 369 |
| 70 | evaluat*.mp,kw. | 394162 |
| 71 | exam*.mp,kw. | 343885 |
| 72 | focus group*.mp,kw. | 6534 |
| 73 | (health adj2 determinant*).mp. | 1535 |
| 74 | HRQoL.mp,kw. | 2111 |
| 75 | index*.mp,kw. | 105173 |
| 76 | indices.mp,kw. | 16842 |
| 77 | indicator?.mp. | 30552 |
| 78 | interview*.mp,kw. | 38797 |
| 79 | instrument?.mp,kw. | 27063 |
| 80 | inventories.mp,kw. | 785 |
| 81 | inventory.mp,kw. | 9789 |
| 82 | measur*.mp,kw. | 443361 |
| 83 | metric?.mp,kw. | 11627 |
| 84 | narrati*.mp,kw. | 7002 |
| 85 | nomogram*3.mp,kw. | 909 |
| 86 | patient analys#s.mp,kw. | 71 |
| 87 | poll?.mp,kw. | 299 |
| 88 | QoL.mp,kw. | 5065 |
| 89 | quality of life.mp,kw. | 37263 |
| 90 | questionnaire?.mp,kw. | 61184 |
| 91 | rating?.mp,kw. | 17616 |
| 92 | (scale or scales).mp,kw. | 138357 |
| 93 | screening?.mp,kw. | 59628 |
| 94 | (score or scores or scored or scoring?).mp,kw. | 117182 |
| 95 | status exam*.mp,kw. | 285 |
| 96 | survey*.mp,kw. | 72459 |
| 97 | (test or tests).mp,kw. | 215779 |
| 98 | (tool? or toolkit? or tool-kit?).mp,kw. | 102760 |
| 99 | psychological tests/ or trail making test/ | 0 |
| 100 | exp Psychiatric Status Rating Scales/ | 0 |
| 101 | Geriatric Assessment/ | 0 |
| 102 | Mass Screening/ | 0 |
| 103 | (battery or batteries).mp,kw. | 15565 |
| 104 | "diagnostic and statistical manual of mental disorders".mp,kw,kf. | 1145 |
| 105 | "diagnostic & statistical manual of mental disorders".mp,kw,kf. | 5 |
| 106 | DSM.mp,kw,kf. | 4858 |
| 107 | (DSM1 or DSM2 or DSM3 or DSM4 or DSM5).mp,kw,kf. | 28 |
| 108 | or/49-107 [ Assessment or Measurement or Scales or Indexes & related terms; QoL & HRQoL added ] | 1445054 |
| 109 | 48 and 108 [ Delirium + Assessment ] | 49775 |
| 110 | Preoperative Care/ | 0 |
| 111 | preoperative period/ | 0 |
| 112 | (preoperat* or pre-operat*).mp,kw. | 32834 |
| 113 | (before adj2 surg*).mp,kw. | 5102 |
| 114 | (before adj2 procedur*).mp,kw. | 768 |
| 115 | (before adj2 operat????).mp,kw. | 1225 |
| 116 | (prior adj2 procedur*).mp,kw. | 231 |
| 117 | (prior adj2 operat????).mp,kw. | 194 |
| 118 | (prior adj1 surg*).mp,kw. | 441 |
| 119 | pre-procedur*.mp,kw. | 428 |
| 120 | preprocedur*.mp,kw. | 411 |
| 121 | preintervention*.mp,kw. | 449 |
| 122 | pre-intervention*.mp,kw. | 812 |
| 123 | pre-surg*.mp,kw. | 649 |
| 124 | "pre- and post-surgery".mp,kw. | 84 |
| 125 | "pre- and post-surgical".mp,kw. | 46 |
| 126 | "pre- and postsurgery".mp,kw. | 24 |
| 127 | "pre- and postsurgical".mp,kw. | 41 |
| 128 | or/110-127 [ Preoperative & related terms ] | 41003 |
| 129 | 109 and 128 [ Delirium + Assessment + Preoperative ] | 618 |
| 130 | exp Surgical Procedures, Operative/ | 1 |
| 131 | su.fs. ["surgery" floating subheading ] | 1 |
| 132 | exp postoperative care/ | 0 |
| 133 | exp Postoperative Period/ | 0 |
| 134 | exp Postoperative Complications/ | 0 |
| 135 | "after surgery".mp,kw. | 15903 |
| 136 | "after surgical*".mp,kw. | 2795 |
| 137 | (following adj2 (surgery or surgeries or surgical)).mp,kw. | 6157 |
| 138 | (postoperat* or post-operat*).mp,kw. | 62099 |
| 139 | post-intervention*.mp,kw. | 2650 |
| 140 | postintervention*.mp,kw. | 1122 |
| 141 | Post-procedur*.mp,kw. | 1186 |
| 142 | postprocedur*.mp,kw. | 746 |
| 143 | post-surg*.mp,kw. | 2743 |
| 144 | postsurg*.mp,kw. | 2095 |
| 145 | or/130-144 [ Postoperative Care/Period/Complications ] | 83688 |
| 146 | 129 and 145 [ Delirium + Assessment + Preoperative + Postoperative ] | 446 |
| 147 | Randomized controlled trial.pt. | 279 |
| 148 | exp Randomized controlled trial/ | 279 |
| 149 | exp Randomized Controlled Trials as Topic/ | 0 |
| 150 | Pragmatic Clinical Trial.pt. | 0 |
| 151 | Pragmatic Clinical Trial/ | 0 |
| 152 | Pragmatic Clinical Trials As Topic/ | 0 |
| 153 | (pragmatic adj2 (trial? or study or studies)).mp,kw. | 411 |
| 154 | random*.mp,kw. | 141241 |
| 155 | Double-Blind Method/ | 0 |
| 156 | ((single or double or triple or treble) adj3 (blind* or mask*)).mp,kw. | 13965 |
| 157 | doubleblind*.mp,kw. | 23 |
| 158 | Placebos/ | 0 |
| 159 | Placebo*.mp,kw. | 17309 |
| 160 | cohort*.mp,kw. | 68837 |
| 161 | evaluation studies.pt. | 26 |
| 162 | (evaluation adj1 (study or studies)).mp,kw. | 897 |
| 163 | validation studies.pt. | 0 |
| 164 | (validation adj1 (study or studies)).mp,kw. | 2285 |
| 165 | clinical trial.pt. | 366 |
| 166 | Controlled Clinical Trial.pt. | 20 |
| 167 | Controlled Clinical Trial/ | 20 |
| 168 | Controlled Clinical Trials As Topic/ | 0 |
| 169 | (controlled adj1 clinical adj2 (trial? or study or studies)).mp,kw. | 3557 |
| 170 | meta-analysis.pt. | 36 |
| 171 | meta-analysis/ | 36 |
| 172 | meta-analysis as topic/ | 1 |
| 173 | (meta-anal* or metanal* or metaanal*).mp,kw. | 25273 |
| 174 | (systematic adj4 (review or reviews or overview or overviews)).mp,kw. | 29434 |
| 175 | (overview? adj4 (review or reviews)).mp,kw. | 3337 |
| 176 | exp Cohort Studies/ | 0 |
| 177 | exp Case-Control Studies/ | 0 |
| 178 | (case control* adj2 (study or studies)).mp,kw. | 11499 |
| 179 | Longitudinal Studies/ | 0 |
| 180 | (longitudinal* adj2 (study or studies)).mp,kw. | 10594 |
| 181 | Prospective Studies/ | 0 |
| 182 | (prospective* adj2 (study or studies)).mp,kw. | 30940 |
| 183 | Cross-Sectional Studies/ | 0 |
| 184 | (cross-sectional* adj2 (study or studies)).mp,kw. | 30676 |
| 185 | case series.mp,kw. | 11121 |
| 186 | Retrospective Studies/ [added May 9 ] | 0 |
| 187 | (retrospective* adj2 (study or studies)).mp,kw. [ added May 9 ] | 32052 |
| 188 | "sensitivity and specificity"/ or "predictive value of tests"/ or roc curve/ [ added May 9 ] | 0 |
| 189 | or/147-188 [ HEDGE - Therapy Question with All Types of Studies ] | 329241 |
| 190 | 146 and 189 [ Delirium + Assessment + Preoperative + Postoperative + Studies ] | 195 |
| 191 | exp Biomarkers/ | 0 |
| 192 | biomarker?.mp. | 41793 |
| 193 | bio-marker?.mp. | 126 |
| 194 | (marker or markers).mp. | 75316 |
| 195 | exp proteomics/ or proteogenomics/ | 0 |
| 196 | proteomic??.mp. | 10299 |
| 197 | proteogenomic??.mp. | 102 |
| 198 | exp Endophenotypes/ | 0 |
| 199 | endophenotyp???.mp. | 552 |
| 200 | exp Genetic Markers/ | 0 |
| 201 | or/191-200 [ Biomarkers & related terms ] | 117269 |
| 202 | 48 and 128 and 145 and 189 and 201 [ Delirium + Assessment + Preoperative + Postoperative + Studies + Biomarkers ] | 7 |

# Embase

Embase Classic+Embase 1947 to 2018 June 21

| **#** | **Searches** | **Results** |
| --- | --- | --- |
| 1 | (postoperative adj1 delirium).mp,kw. | 2165 |
| 2 | (post-operative adj1 delirium).mp,kw. | 236 |
| 3 | Emergence Delirium/ [ New MeSH as of 2017 ] | 40 |
| 4 | cognition disorders/ or mild cognitive impairment/ | 45580 |
| 5 | (cognitiv* adj2 impair*).mp,kw. | 96343 |
| 6 | (disorder* adj2 cognit*).mp,kw. | 12346 |
| 7 | (cognitiv* adj2 status).mp,kw. | 7942 |
| 8 | (cognitiv* adj2 dysfunc*).mp,kw. | 20828 |
| 9 | (cognitiv* adj2 function*).mp,kw. | 84047 |
| 10 | dyscognitiv*.mp,kw. | 150 |
| 11 | cognitive*.mp,kw. | 490720 |
| 12 | Cognition/ | 214060 |
| 13 | cognition*.mp,kw. | 260930 |
| 14 | Confusion/ | 27560 |
| 15 | (confuse? or confusing or confusion*).mp,kw. | 98766 |
| 16 | Delirium/ | 24518 |
| 17 | delirium?.mp,kw. | 31255 |
| 18 | delirius.mp,kw. | 4 |
| 19 | delirious*.mp,kw. | 2403 |
| 20 | Hallucinations/ | 14408 |
| 21 | hallucinat*.mp,kw. | 40356 |
| 22 | Dementia/ | 107780 |
| 23 | dementia?.mp,kw. | 177922 |
| 24 | demented??.mp,kw. | 12192 |
| 25 | dementat*.mp,kw. | 4 |
| 26 | demenc???.mp,kw. | 2259 |
| 27 | (intellectual* adj2 declin*).mp,kw. | 449 |
| 28 | amentia???.mp,kw. | 236 |
| 29 | (mental* adj2 deteriorat*).mp,kw. | 7524 |
| 30 | (mental* adj2 acuit*).mp,kw. | 165 |
| 31 | (post-an?esth* adj1 excit*).mp,kw. | 5 |
| 32 | (postan?esth* adj1 excit*).mp,kw. | 18 |
| 33 | (emergence adj1 agitat*).mp,kw. | 503 |
| 34 | (emergence adj1 excit*).mp,kw. | 15 |
| 35 | emergence agitation/ [Embase] | 209 |
| 36 | postoperative cognitive dysfunction/ | 865 |
| 37 | postoperative delirium/ | 1506 |
| 38 | (acute?? adj1 confusion?? adj1 state?).mp,kw. | 745 |
| 39 | (toxic-metabolic adj1 encephalopath*).mp,kw. | 95 |
| 40 | (acute adj1 brain adj1 syndrome?).mp,kw. | 120 |
| 41 | (acute adj1 brain adj1 fail*).mp,kw. | 26 |
| 42 | (acute adj1 organ?? adj1 psychos#s).mp,kw. | 32 |
| 43 | (acute adj1 organ?? adj1 brain syndrome?).mp,kw. | 61 |
| 44 | (ICU adj2 psychos#s).mp,kw. | 46 |
| 45 | (Intensive Care adj3 psychos#s).mp,kw. | 329 |
| 46 | (metabolic adj1 encephalopath*).mp,kw. | 1989 |
| 47 | pseudosenilit*.mp,kw. | 2 |
| 48 | pseudo-senilit*.mp,kw. | 2 |
| 49 | (reversab* adj2 dementia?).mp,kw. | 2 |
| 50 | (reversib* adj2 dementia?).mp,kw. | 347 |
| 51 | (toxic adj2 psychos#s).mp,kw. | 688 |
| 52 | or/1-51 [ ~~ Cognition / Cognitive Impairment / Confusion / Delirium ~~ ] | 825680 |
| 53 | Symptom Assessment/ | 5594 |
| 54 | Algorithms/ | 161329 |
| 55 | [Data Collection/ ==> do not use in Embase] | 0 |
| 56 | [Focus Groups/ ==> do not use in Embase ] | 0 |
| 57 | Health Care Surveys/ | 9006 |
| 58 | Health Impact Assessment/ | 3468 |
| 59 | Health Status Indicators/ | 1692 |
| 60 | Health Surveys/ | 168605 |
| 61 | Interviews As Topic/ | 126487 |
| 62 | Narration/ | 13229 |
| 63 | Patient Acuity/ | 653 |
| 64 | Patient Reported Outcome Measures/ | 133 |
| 65 | "Quality of Life"/ | 391036 |
| 66 | "Severity Of Illness Index"/ | 12102 |
| 67 | Sickness Impact Profile/ | 2216 |
| 68 | "Surveys and Questionnaires"/ | 585999 |
| 69 | algorithm*.mp,kw. | 347819 |
| 70 | assess*.mp,kw. | 4399379 |
| 71 | audit?.mp,kw. | 78481 |
| 72 | checklist?.mp,kw. | 48609 |
| 73 | check-list?.mp,kw. | 4497 |
| 74 | evaluat*.mp,kw. | 4465998 |
| 75 | exam*.mp,kw. | 4149960 |
| 76 | focus group*.mp,kw. | 45370 |
| 77 | (health adj2 determinant*).mp. | 10541 |
| 78 | HRQoL.mp,kw. | 21167 |
| 79 | index*.mp,kw. | 1072279 |
| 80 | indices.mp,kw. | 180250 |
| 81 | indicator?.mp. | 342620 |
| 82 | interview*.mp,kw. | 425778 |
| 83 | instrument?.mp,kw. | 274766 |
| 84 | inventories.mp,kw. | 21036 |
| 85 | inventory.mp,kw. | 114512 |
| 86 | measur*.mp,kw. | 4181307 |
| 87 | metric?.mp,kw. | 61199 |
| 88 | narrati*.mp,kw. | 35304 |
| 89 | nomogram*3.mp,kw. | 11851 |
| 90 | patient analys#s.mp,kw. | 1187 |
| 91 | poll?.mp,kw. | 3788 |
| 92 | QoL.mp,kw. | 59115 |
| 93 | quality of life.mp,kw. | 488749 |
| 94 | questionnaire?.mp,kw. | 805492 |
| 95 | rating?.mp,kw. | 290394 |
| 96 | (scale or scales).mp,kw. | 1034766 |
| 97 | screening?.mp,kw. | 933234 |
| 98 | (score or scores or scored or scoring?).mp,kw. | 1315806 |
| 99 | status exam*.mp,kw. | 3331 |
| 100 | survey*.mp,kw. | 1372915 |
| 101 | (test or tests).mp,kw. | 3238994 |
| 102 | (tool? or toolkit? or tool-kit?).mp,kw. | 811250 |
| 103 | psychological tests/ or trail making test/ [ added May 9 ] | 39537 |
| 104 | exp Psychiatric Status Rating Scales/ [added May 9 ] | 19361 |
| 105 | Geriatric Assessment/ [ added May 9 ] | 13691 |
| 106 | Mass Screening/ [ added May 9 ] | 55672 |
| 107 | (battery or batteries).mp,kw. [ added May 9 ] | 69247 |
| 108 | data collection method/ [Embase] | 4375 |
| 109 | disease activity score/ | 4007 |
| 110 | interview/ | 177422 |
| 111 | structured interview/ | 11926 |
| 112 | telephone interview/ | 8305 |
| 113 | narrative/ | 7448 |
| 114 | patient-reported outcome/ | 8713 |
| 115 | general health status assessment/ | 533 |
| 116 | psychological rating scale/ | 19361 |
| 117 | "diagnostic and statistical manual of mental disorders".mp,kw. | 28144 |
| 118 | "diagnostic & statistical manual of mental disorders".mp,kw. | 50 |
| 119 | DSM.mp,kw. | 76365 |
| 120 | (DSM1 or DSM2 or DSM3 or DSM4 or DSM5).mp,kw. | 200 |
| 121 | or/53-120 [ Assessment or Measurement or Scales or Indexes & related terms; QoL & HRQoL added ] | 16102062 |
| 122 | 52 and 121 [ Delirium + Assessment ] | 587148 |
| 123 | Preoperative Care/ | 39840 |
| 124 | preoperative period/ | 44896 |
| 125 | (preoperat* or pre-operat*).mp,kw. | 472796 |
| 126 | (before adj2 surg*).mp,kw. | 66463 |
| 127 | (before adj2 procedur*).mp,kw. | 11204 |
| 128 | (before adj2 operat????).mp,kw. | 26471 |
| 129 | (prior adj2 procedur*).mp,kw. | 3516 |
| 130 | (prior adj2 operat????).mp,kw. | 4457 |
| 131 | (prior adj1 surg*).mp,kw. | 6191 |
| 132 | pre-procedur*.mp,kw. | 6268 |
| 133 | preprocedur*.mp,kw. | 4792 |
| 134 | preintervention*.mp,kw. | 3846 |
| 135 | pre-intervention*.mp,kw. | 6545 |
| 136 | pre-surg*.mp,kw. | 7135 |
| 137 | "pre- and post-surgery".mp,kw. | 1037 |
| 138 | "pre- and post-surgical".mp,kw. | 653 |
| 139 | "pre- and postsurgery".mp,kw. | 267 |
| 140 | "pre- and postsurgical".mp,kw. | 451 |
| 141 | or/123-140 [ Preoperative & related terms ] | 577595 |
| 142 | 122 and 141 [ Delirium + Assessment + Preoperative ] | 9242 |
| 143 | exp Surgical Procedures, Operative/ | 4683065 |
| 144 | su.fs. ["surgery" floating subheading ] | 1992972 |
| 145 | exp postoperative care/ | 85615 |
| 146 | exp Postoperative Period/ | 465056 |
| 147 | postoperative complication/ | 319785 |
| 148 | "after surgery".mp,kw. | 185925 |
| 149 | "after surgical*".mp,kw. | 37276 |
| 150 | (following adj2 (surgery or surgeries or surgical)).mp,kw. | 65953 |
| 151 | (postoperat* or post-operat*).mp,kw. | 1078944 |
| 152 | post-intervention*.mp,kw. | 19033 |
| 153 | postintervention*.mp,kw. | 8342 |
| 154 | Post-procedur*.mp,kw. | 16765 |
| 155 | postprocedur*.mp,kw. | 8754 |
| 156 | post-surg*.mp,kw. | 28220 |
| 157 | postsurg*.mp,kw. | 21782 |
| 158 | exp surgery/ [Embase] | 4683065 |
| 159 | or/143-158 [ Postoperative Care/Period/Complications ] | 5303303 |
| 160 | 142 and 159 [ Delirium + Assessment + Preoperative + Postoperative ] | 8529 |
| 161 | exp Randomized controlled trial/ | 507508 |
| 162 | exp Randomized Controlled Trials as Topic/ | 147510 |
| 163 | Pragmatic Clinical Trial/ | 417981 |
| 164 | Pragmatic Clinical Trials As Topic/ | 147510 |
| 165 | (pragmatic adj2 (trial? or study or studies)).mp,kw. | 2718 |
| 166 | random*.mp,kw. | 1528133 |
| 167 | Double-Blind Method/ | 130117 |
| 168 | ((single or double or triple or treble) adj3 (blind* or mask*)).mp,kw. | 280445 |
| 169 | doubleblind*.mp,kw. | 2923 |
| 170 | Placebos/ | 274925 |
| 171 | Placebo*.mp,kw. | 426045 |
| 172 | cohort*.mp,kw. | 843863 |
| 173 | (evaluation adj1 (study or studies)).mp,kw. | 41272 |
| 174 | (validation adj1 (study or studies)).mp,kw. | 80125 |
| 175 | Controlled Clinical Trial/ | 461218 |
| 176 | Controlled Clinical Trials As Topic/ | 8289 |
| 177 | (controlled adj1 clinical adj2 (trial? or study or studies)).mp,kw. | 493317 |
| 178 | meta-analysis/ | 146146 |
| 179 | meta-analysis as topic/ | 26114 |
| 180 | (meta-anal* or metanal* or metaanal*).mp,kw. | 237467 |
| 181 | (systematic adj4 (review or reviews or overview or overviews)).mp,kw. | 240757 |
| 182 | (overview? adj4 (review or reviews)).mp,kw. | 15581 |
| 183 | exp Cohort Studies/ | 381804 |
| 184 | exp Case-Control Studies/ | 144959 |
| 185 | (case control* adj2 (study or studies)).mp,kw. | 186748 |
| 186 | Longitudinal Studies/ | 97849 |
| 187 | (longitudinal* adj2 (study or studies)).mp,kw. | 159824 |
| 188 | Prospective Studies/ | 360662 |
| 189 | (prospective* adj2 (study or studies)).mp,kw. | 632015 |
| 190 | Cross-Sectional Studies/ | 140707 |
| 191 | (cross-sectional* adj2 (study or studies)).mp,kw. | 315041 |
| 192 | case series.mp,kw. | 82431 |
| 193 | Retrospective Studies/ [added May 9 ] | 447024 |
| 194 | (retrospective* adj2 (study or studies)).mp,kw. [ added May 9 ] | 757184 |
| 195 | "sensitivity and specificity"/ or "predictive value of tests"/ or roc curve/ [ added May 9 ] | 360575 |
| 196 | ct.fs. [clinical trial - Embase] | 594071 |
| 197 | exp clinical trial/ or "clinical trial (topic)"/ | 1422219 |
| 198 | random sample/ or randomization/ or randomized controlled trial/ or "randomized controlled trial (topic)"/ | 725898 |
| 199 | cohort analysis/ | 381804 |
| 200 | case control study/ or hospital based case control study/ | 129945 |
| 201 | longitudinal study/ | 114616 |
| 202 | prospective study/ | 457324 |
| 203 | retrospective study/ | 663586 |
| 204 | "sensitivity and specificity"/ | 297679 |
| 205 | predictive validity/ or predictive value/ | 138229 |
| 206 | receiver operating characteristic/ | 91536 |
| 207 | cross-sectional study/ | 257745 |
| 208 | exp meta analysis/ or "meta analysis (topic)"/ | 180516 |
| 209 | "systematic review"/ or "systematic review (topic)"/ | 190614 |
| 210 | or/161-209 [ Studies ] | 5094478 |
| 211 | 160 and 210 [ Delirium + Assessment + Preoperative + Postoperative + Studies ] | 3857 |
| 212 | limit 211 to english | 3693 |
| 213 | (exp animals/ or exp animal experimentation/ or nonhuman/) not ((exp animals/ or exp animal experimentation/ or nonhuman/) and exp human/) | 6746637 |
| 214 | 212 not 213 | 3657 |
| 215 | limit 212 to human | 3552 |
| 216 | 214 or 215 | 3657 |
| 217 | limit 216 to (books or "book review" or chapter or conference abstract or "conference review") | 838 |
| 218 | 216 not 217 | 2819 |
| 219 | remove duplicates from 218 | 2731 |
| 220 | exp Biomarkers/ | 236129 |
| 221 | biomarker?.mp. | 314705 |
| 222 | bio-marker?.mp. | 1523 |
| 223 | (marker or markers).mp. | 1191979 |
| 224 | exp proteomics/ or proteogenomics/ | 79273 |
| 225 | proteomic??.mp. | 107781 |
| 226 | proteogenomic??.mp. | 621 |
| 227 | exp Endophenotypes/ | 3309 |
| 228 | endophenotyp???.mp. | 6770 |
| 229 | exp Genetic Markers/ | 83613 |
| 230 | biological marker/ | 236129 |
| 231 | proteome/ or exp proteomics/ | 96467 |
| 232 | proteogenomics/ | 199 |
| 233 | endophenotype/ | 3309 |
| 234 | exp genetic marker/ | 83613 |
| 235 | or/220-234 [ Biomarkers & related terms ] | 1403968 |
| 236 | 219 and 235 [ Delirium + Assessment + Preoperative + Postoperative + Studies + Biomarkers ] | 106 |

# CCTR

EBM Reviews - Cochrane Central Register of Controlled Trials May 2018

| **#** | **Searches** | **Results** |
| --- | --- | --- |
| 1 | (postoperative adj1 delirium).mp,kw. | 324 |
| 2 | (post-operative adj1 delirium).mp,kw. | 56 |
| 3 | Emergence Delirium/ [ New MeSH as of 2017 ] | 32 |
| 4 | cognition disorders/ or mild cognitive impairment/ | 3224 |
| 5 | (cognitiv* adj2 impair*).mp,kw. | 6396 |
| 6 | (disorder* adj2 cognit*).mp,kw. | 4107 |
| 7 | (cognitiv* adj2 status).mp,kw. | 715 |
| 8 | (cognitiv* adj2 dysfunc*).mp,kw. | 1875 |
| 9 | (cognitiv* adj2 function*).mp,kw. | 9841 |
| 10 | dyscognitiv*.mp,kw. | 2 |
| 11 | cognitive*.mp,kw. | 45193 |
| 12 | Cognition/ | 6518 |
| 13 | cognition*.mp,kw. | 18705 |
| 14 | Confusion/ | 133 |
| 15 | (confuse? or confusing or confusion*).mp,kw. | 2145 |
| 16 | Delirium/ | 430 |
| 17 | delirium?.mp,kw. | 1884 |
| 18 | delirius.mp,kw. | 0 |
| 19 | delirious*.mp,kw. | 87 |
| 20 | Hallucinations/ | 295 |
| 21 | hallucinat*.mp,kw. | 1515 |
| 22 | Dementia/ | 1708 |
| 23 | dementia?.mp,kw. | 8534 |
| 24 | demented??.mp,kw. | 549 |
| 25 | dementat*.mp,kw. | 0 |
| 26 | demenc???.mp,kw. | 28 |
| 27 | (intellectual* adj2 declin*).mp,kw. | 10 |
| 28 | amentia???.mp,kw. | 1 |
| 29 | (mental* adj2 deteriorat*).mp,kw. | 218 |
| 30 | (mental* adj2 acuit*).mp,kw. | 18 |
| 31 | (post-an?esth* adj1 excit*).mp,kw. | 1 |
| 32 | (postan?esth* adj1 excit*).mp,kw. | 3 |
| 33 | (emergence adj1 agitat*).mp,kw. | 365 |
| 34 | (emergence adj1 excit*).mp,kw. | 4 |
| 35 | emergence agitation/ [Embase] | 0 |
| 36 | postoperative cognitive dysfunction/ | 1 |
| 37 | postoperative delirium/ | 0 |
| 38 | (acute?? adj1 confusion?? adj1 state?).mp,kw. | 8 |
| 39 | (toxic-metabolic adj1 encephalopath*).mp,kw. | 3 |
| 40 | (acute adj1 brain adj1 syndrome?).mp,kw. | 2 |
| 41 | (acute adj1 brain adj1 fail*).mp,kw. | 2 |
| 42 | (acute adj1 organ?? adj1 psychos#s).mp,kw. | 0 |
| 43 | (acute adj1 organ?? adj1 brain syndrome?).mp,kw. | 1 |
| 44 | (ICU adj2 psychos#s).mp,kw. | 0 |
| 45 | (Intensive Care adj3 psychos#s).mp,kw. | 22 |
| 46 | (metabolic adj1 encephalopath*).mp,kw. | 15 |
| 47 | pseudosenilit*.mp,kw. | 0 |
| 48 | pseudo-senilit*.mp,kw. | 0 |
| 49 | (reversab* adj2 dementia?).mp,kw. | 0 |
| 50 | (reversib* adj2 dementia?).mp,kw. | 6 |
| 51 | (toxic adj2 psychos#s).mp,kw. [ added June 20 2018 ] | 5 |
| 52 | or/1-51 [ ~~ Cognition / Cognitive Impairment / Confusion / Delirium ~~ ] | 58919 |
| 53 | Symptom Assessment/ | 161 |
| 54 | Algorithms/ | 3278 |
| 55 | Data Collection/ | 1199 |
| 56 | Focus Groups/ | 503 |
| 57 | Health Care Surveys/ | 324 |
| 58 | Health Impact Assessment/ | 6 |
| 59 | Health Status Indicators/ | 958 |
| 60 | Health Surveys/ | 838 |
| 61 | Interviews As Topic/ | 1698 |
| 62 | Narration/ | 168 |
| 63 | Patient Acuity/ | 50 |
| 64 | Patient Reported Outcome Measures/ | 161 |
| 65 | "Quality of Life"/ | 19502 |
| 66 | "Severity Of Illness Index"/ | 17467 |
| 67 | Sickness Impact Profile/ | 520 |
| 68 | "Surveys and Questionnaires"/ | 22881 |
| 69 | algorithm*.mp,kw. | 9583 |
| 70 | assess*.mp,kw. | 342872 |
| 71 | audit?.mp,kw. | 2193 |
| 72 | checklist?.mp,kw. | 3856 |
| 73 | check-list?.mp,kw. | 457 |
| 74 | evaluat*.mp,kw. | 346046 |
| 75 | exam*.mp,kw. | 139115 |
| 76 | focus group*.mp,kw. | 1948 |
| 77 | (health adj2 determinant*).mp. | 189 |
| 78 | HRQoL.mp,kw. | 3066 |
| 79 | index*.mp,kw. | 102230 |
| 80 | indices.mp,kw. | 11204 |
| 81 | indicator?.mp. | 11851 |
| 82 | interview*.mp,kw. | 21394 |
| 83 | instrument?.mp,kw. | 11261 |
| 84 | inventories.mp,kw. | 1284 |
| 85 | inventory.mp,kw. | 12845 |
| 86 | measur*.mp,kw. | 305194 |
| 87 | metric?.mp,kw. | 2035 |
| 88 | narrati*.mp,kw. | 1100 |
| 89 | nomogram*3.mp,kw. | 503 |
| 90 | patient analys#s.mp,kw. | 201 |
| 91 | poll?.mp,kw. | 21 |
| 92 | QoL.mp,kw. | 9603 |
| 93 | quality of life.mp,kw. | 60753 |
| 94 | questionnaire?.mp,kw. | 71617 |
| 95 | rating?.mp,kw. | 45304 |
| 96 | (scale or scales).mp,kw. | 113004 |
| 97 | screening?.mp,kw. | 26511 |
| 98 | (score or scores or scored or scoring?).mp,kw. | 162481 |
| 99 | status exam*.mp,kw. | 381 |
| 100 | survey*.mp,kw. | 42027 |
| 101 | (test or tests).mp,kw. | 206655 |
| 102 | (tool? or toolkit? or tool-kit?).mp,kw. | 21414 |
| 103 | psychological tests/ or trail making test/ [ added May 9 ] | 1825 |
| 104 | exp Psychiatric Status Rating Scales/ [added May 9 ] | 10067 |
| 105 | Geriatric Assessment/ [ added May 9 ] | 1317 |
| 106 | Mass Screening/ [ added May 9 ] | 2823 |
| 107 | (battery or batteries).mp,kw. [ added May 9 ] | 5435 |
| 108 | data collection method/ [Embase] | 0 |
| 109 | disease activity score/ | 0 |
| 110 | interview/ | 8 |
| 111 | structured interview/ | 0 |
| 112 | telephone interview/ | 1 |
| 113 | narrative/ | 0 |
| 114 | patient-reported outcome/ | 0 |
| 115 | general health status assessment/ | 0 |
| 116 | psychological rating scale/ | 0 |
| 117 | "diagnostic and statistical manual of mental disorders".mp,kw,kf. | 2010 |
| 118 | "diagnostic & statistical manual of mental disorders".mp,kw,kf. | 2004 |
| 119 | DSM.mp,kw,kf. | 7167 |
| 120 | (DSM1 or DSM2 or DSM3 or DSM4 or DSM5).mp,kw,kf. | 8 |
| 121 | or/53-120 [ Assessment or Measurement or Scales or Indexes & related terms; QoL & HRQoL added ] | 834749 |
| 122 | 52 and 121 [ Delirium + Assessment ] | 49423 |
| 123 | Preoperative Care/ | 3812 |
| 124 | preoperative period/ | 214 |
| 125 | (preoperat* or pre-operat*).mp,kw. | 30634 |
| 126 | (before adj2 surg*).mp,kw. | 153710 |
| 127 | (before adj2 procedur*).mp,kw. | 153369 |
| 128 | (before adj2 operat????).mp,kw. | 63249 |
| 129 | (prior adj2 procedur*).mp,kw. | 702 |
| 130 | (prior adj2 operat????).mp,kw. | 439 |
| 131 | (prior adj1 surg*).mp,kw. | 2453 |
| 132 | pre-procedur*.mp,kw. | 512 |
| 133 | preprocedur*.mp,kw. | 444 |
| 134 | preintervention*.mp,kw. | 902 |
| 135 | pre-intervention*.mp,kw. | 1361 |
| 136 | pre-surg*.mp,kw. | 470 |
| 137 | "pre- and post-surgery".mp,kw. | 64 |
| 138 | "pre- and post-surgical".mp,kw. | 28 |
| 139 | "pre- and postsurgery".mp,kw. | 27 |
| 140 | "pre- and postsurgical".mp,kw. | 16 |
| 141 | or/123-140 [ Preoperative & related terms ] | 285025 |
| 142 | 122 and 141 [ Delirium + Assessment + Preoperative ] | 10175 |
| 143 | exp Surgical Procedures, Operative/ | 105067 |
| 144 | su.fs. ["surgery" floating subheading ] | 52213 |
| 145 | exp postoperative care/ | 4156 |
| 146 | exp Postoperative Period/ | 5306 |
| 147 | postoperative complication/ | 1 |
| 148 | "after surgery".mp,kw. | 123243 |
| 149 | "after surgical*".mp,kw. | 70346 |
| 150 | (following adj2 (surgery or surgeries or surgical)).mp,kw. | 149261 |
| 151 | (postoperat* or post-operat*).mp,kw. | 90880 |
| 152 | post-intervention*.mp,kw. | 6511 |
| 153 | postintervention*.mp,kw. | 2859 |
| 154 | Post-procedur*.mp,kw. | 1512 |
| 155 | postprocedur*.mp,kw. | 933 |
| 156 | post-surg*.mp,kw. | 2481 |
| 157 | postsurg*.mp,kw. | 1990 |
| 158 | exp surgery/ [Embase] | 321 |
| 159 | or/143-158 [ Postoperative Care/Period/Complications ] | 229192 |
| 160 | 142 and 159 [ Delirium + Assessment + Preoperative + Postoperative ] | 3624 |
| 161 | exp Biomarkers/ | 6757 |
| 162 | biomarker?.mp. | 27247 |
| 163 | bio-marker?.mp. | 63 |
| 164 | (marker or markers).mp. | 38842 |
| 165 | exp proteomics/ or proteogenomics/ | 85 |
| 166 | proteomic??.mp. | 536 |
| 167 | proteogenomic??.mp. | 2 |
| 168 | exp Endophenotypes/ | 10 |
| 169 | endophenotyp???.mp. | 87 |
| 170 | exp Genetic Markers/ | 276 |
| 171 | biological marker/ | 2 |
| 172 | proteome/ or exp proteomics/ | 117 |
| 173 | proteogenomics/ | 0 |
| 174 | endophenotype/ | 10 |
| 175 | exp genetic marker/ | 276 |
| 176 | or/161-175 [ Biomarkers & related terms ] | 57259 |
| 177 | 52 and 141 and 159 and 176 | 162 |
| 178 | limit 177 to english language | 131 |
| 179 | conferenc*.so. | 56535 |
| 180 | 178 not 179 | 122 |
| 181 | limit 180 to medline records | 69 |
| 182 | limit 180 to embase records | 52 |
| 183 | 181 or 182 | 121 |
| 184 | 180 not 183 | 1 |

# CDSR

EBM Reviews - Cochrane Database of Systematic Reviews 2005 to June 20, 2018

| **#** | **Searches** | **Results** |
| --- | --- | --- |
| 1 | (postoperative adj1 delirium).ti,ab. | 3 |
| 2 | (post-operative adj1 delirium).ti,ab. | 1 |
| 3 | (cognitiv* adj2 impair*).ti,ab. | 107 |
| 4 | (disorder* adj2 cognit*).ti,ab. | 3 |
| 5 | (cognitiv* adj2 status).ti,ab. | 7 |
| 6 | (cognitiv* adj2 dysfunc*).ti,ab. | 11 |
| 7 | (cognitiv* adj2 function*).ti,ab. | 115 |
| 8 | dyscognitiv*.ti,ab. | 0 |
| 9 | cognitive*.ti,ab. | 460 |
| 10 | cognition*.ti,ab. | 87 |
| 11 | (confuse? or confusing or confusion*).ti,ab. | 24 |
| 12 | delirium?.ti,ab. | 24 |
| 13 | delirius.ti,ab. | 0 |
| 14 | delirious*.ti,ab. | 0 |
| 15 | hallucinat*.ti,ab. | 17 |
| 16 | dementia?.ti,ab. | 205 |
| 17 | demented??.ti,ab. | 8 |
| 18 | dementat*.ti,ab. | 0 |
| 19 | demenc???.ti,ab. | 0 |
| 20 | (intellectual* adj2 declin*).ti,ab. | 0 |
| 21 | amentia???.ti,ab. | 0 |
| 22 | (mental* adj2 deteriorat*).ti,ab. | 1 |
| 23 | (mental* adj2 acuit*).ti,ab. | 0 |
| 24 | (post-an?esth* adj1 excit*).ti,ab. | 0 |
| 25 | (postan?esth* adj1 excit*).ti,ab. | 0 |
| 26 | (emergence adj1 agitat*).ti,ab. | 1 |
| 27 | (emergence adj1 excit*).ti,ab. | 0 |
| 28 | (acute?? adj1 confusion?? adj1 state?).ti,ab. | 3 |
| 29 | (toxic-metabolic adj1 encephalopath*).ti,ab. | 0 |
| 30 | (acute adj1 brain adj1 fail*).ti,ab. | 0 |
| 31 | (acute adj1 brain adj1 syndrome?).ti,ab. | 0 |
| 32 | (acute adj1 organ?? adj1 psychos#s).ti,ab. | 0 |
| 33 | (acute adj1 organ?? adj1 brain syndrome?).ti,ab. | 0 |
| 34 | (ICU adj2 psychos#s).ti,ab. | 0 |
| 35 | (Intensive Care adj3 psychos#s).ti,ab. | 0 |
| 36 | (metabolic adj1 encephalopath*).ti,ab. | 0 |
| 37 | pseudosenilit*.ti,ab. | 0 |
| 38 | pseudo-senilit*.ti,ab. | 0 |
| 39 | (reversab* adj2 dementia?).ti,ab. | 0 |
| 40 | (reversib* adj2 dementia?).ti,ab. | 0 |
| 41 | (toxic adj2 psychos#s).ti,ab. | 0 |
| 42 | or/1-41 [ Delirium & related terms ] | 559 |
| 43 | algorithm*.ti,ab. | 22 |
| 44 | assess*.ti,ab. | 7372 |
| 45 | audit?.ti,ab. | 15 |
| 46 | checklist?.ti,ab. | 47 |
| 47 | check-list?.ti,ab. | 4 |
| 48 | evaluat*.ti,ab. | 3310 |
| 49 | exam*.ti,ab. | 1548 |
| 50 | focus group*.ti,ab. | 0 |
| 51 | (health adj2 determinant*).ti,ab. | 4 |
| 52 | HRQoL.ti,ab. | 42 |
| 53 | index*.ti,ab. | 1356 |
| 54 | indices.ti,ab. | 46 |
| 55 | indicator?.mp. | 1424 |
| 56 | interview*.ti,ab. | 42 |
| 57 | instrument?.ti,ab. | 99 |
| 58 | inventories.ti,ab. | 0 |
| 59 | inventory.ti,ab. | 19 |
| 60 | measur*.ti,ab. | 2216 |
| 61 | metric?.ti,ab. | 6 |
| 62 | narrati*.ti,ab. | 217 |
| 63 | nomogram*3.ti,ab. | 1 |
| 64 | patient analys#s.ti,ab. | 1 |
| 65 | poll?.ti,ab. | 0 |
| 66 | QoL.ti,ab. | 76 |
| 67 | quality of life.ti,ab. | 1409 |
| 68 | questionnaire?.ti,ab. | 81 |
| 69 | rating?.ti,ab. | 141 |
| 70 | (scale or scales).ti,ab. | 602 |
| 71 | screening?.ti,ab. | 280 |
| 72 | (score or scores or scored or scoring?).ti,ab. | 726 |
| 73 | status exam*.ti,ab. | 1 |
| 74 | survey*.ti,ab. | 36 |
| 75 | (test or tests).ti,ab. | 639 |
| 76 | (tool? or toolkit? or tool-kit?).ti,ab. | 418 |
| 77 | (battery or batteries).ti,ab. | 5 |
| 78 | "diagnostic and statistical manual of mental disorders".ti,ab. | 17 |
| 79 | "diagnostic & statistical manual of mental disorders".ti,ab. | 17 |
| 80 | DSM.ti,ab. | 34 |
| 81 | (DSM1 or DSM2 or DSM3 or DSM4 or DSM5).ti,ab. | 1 |
| 82 | or/43-81 [ Assessment ] | 8673 |
| 83 | 42 and 82 [ Delirium + Assessment ] | 524 |
| 84 | (preoperat* or pre-operat*).ti,ab. | 106 |
| 85 | (before adj2 surg*).ti,ab. | 1596 |
| 86 | (before adj2 procedur*).ti,ab. | 1015 |
| 87 | (before adj2 operat????).ti,ab. | 470 |
| 88 | (prior adj2 procedur*).ti,ab. | 7 |
| 89 | (prior adj2 operat????).ti,ab. | 1 |
| 90 | (prior adj1 surg*).ti,ab. | 20 |
| 91 | pre-procedur*.ti,ab. | 2 |
| 92 | preprocedur*.ti,ab. | 0 |
| 93 | preintervention*.ti,ab. | 0 |
| 94 | pre-intervention*.ti,ab. | 4 |
| 95 | pre-surg*.ti,ab. | 7 |
| 96 | "pre- and post-surgery".ti,ab. | 1 |
| 97 | "pre- and post-surgical".ti,ab. | 0 |
| 98 | "pre- and postsurgery".ti,ab. | 0 |
| 99 | "pre- and postsurgical".ti,ab. | 0 |
| 100 | or/84-99 [ Preoperative ] | 2371 |
| 101 | 83 and 100 [ Delirium + Assessment + Preoperative ] | 77 |
| 102 | "after surgery".ti,ab. | 1085 |
| 103 | "after surgical*".ti,ab. | 984 |
| 104 | (following adj2 (surgery or surgeries or surgical)).ti,ab. | 1563 |
| 105 | (postoperat* or post-operat*).ti,ab. | 511 |
| 106 | post-intervention*.ti,ab. | 47 |
| 107 | postintervention*.ti,ab. | 5 |
| 108 | Post-procedur*.ti,ab. | 7 |
| 109 | postprocedur*.ti,ab. | 2 |
| 110 | post-surg*.ti,ab. | 33 |
| 111 | postsurg*.ti,ab. | 11 |
| 112 | or/102-111 [ Postoperative ] | 1769 |
| 113 | 101 and 112 [ Delirium + Assessment + Preoperative + Postoperative ] | 31 |
| 114 | limit 113 to full systematic reviews | 31 |
| 115 | remove duplicates from 114 | 31 |
| 116 | biomarker?.ti,ab. | 43 |
| 117 | bio-marker?.ti,ab. | 2 |
| 118 | (marker or markers).ti,ab. | 87 |
| 119 | proteomic??.ti,ab. | 0 |
| 120 | proteogenomic??.ti,ab. | 0 |
| 121 | endophenotyp???.ti,ab. | 0 |
| 122 | or/116-121 [ Biomarkers & related terms ] | 124 |
| 123 | 42 and 100 and 112 and 122 | 1 |
| 124 | limit 123 to full systematic reviews | 1 |

# PsycINFO

PsycINFO 1806 to June Week 3 2018

| **#** | **Searches** | **Results** |
| --- | --- | --- |
| 1 | (postoperative adj1 delirium).mp,kw. | 256 |
| 2 | (post-operative adj1 delirium).mp,kw. | 29 |
| 3 | Delirium/ | 3054 |
| 4 | Cognitive Impairment/ | 32378 |
| 5 | exp Cognition/ | 33363 |
| 6 | Mental Confusion/ | 840 |
| 7 | Consciousness Disturbances/ | 784 |
| 8 | exp Hallucinations/ | 5971 |
| 9 | Dementia/ | 31048 |
| 10 | (cognitiv* adj2 impair*).mp. | 50624 |
| 11 | (disorder* adj2 cognit*).mp. | 5053 |
| 12 | (cognitiv* adj2 status).mp. | 4134 |
| 13 | (cognitiv* adj2 dysfunc*).mp. | 7022 |
| 14 | (cognitiv* adj2 function*).mp. | 41153 |
| 15 | dyscognitiv*.mp. | 24 |
| 16 | cognitive*.mp. | 478903 |
| 17 | cognition*.mp. | 112254 |
| 18 | (confuse? or confusing or confusion*).mp. | 29352 |
| 19 | delirium?.mp. | 6421 |
| 20 | delirius.mp. | 1 |
| 21 | delirious*.mp. | 1086 |
| 22 | hallucinat*.mp. | 15044 |
| 23 | dementia?.mp. | 65118 |
| 24 | demented??.mp. | 5257 |
| 25 | dementat*.mp. | 1 |
| 26 | demenc???.mp. | 761 |
| 27 | (intellectual* adj2 declin*).mp. | 247 |
| 28 | amentia???.mp. | 205 |
| 29 | (mental* adj2 deteriorat*).mp. | 992 |
| 30 | (mental* adj2 acuit*).mp. | 80 |
| 31 | (post-an?esth* adj1 excit*).mp. | 0 |
| 32 | (postan?esth* adj1 excit*).mp. | 0 |
| 33 | (emergence adj1 agitat*).mp. | 6 |
| 34 | (emergence adj1 excit*).mp. | 0 |
| 35 | (acute?? adj1 confusion?? adj1 state?).mp. | 213 |
| 36 | (toxic-metabolic adj1 encephalopath*).mp. | 11 |
| 37 | (acute adj1 brain adj1 syndrome?).mp. | 42 |
| 38 | (acute adj1 brain adj1 fail*).mp. | 12 |
| 39 | (acute adj1 organ?? adj1 psychos#s).mp. | 13 |
| 40 | (acute adj1 organ?? adj1 brain syndrome?).mp. | 20 |
| 41 | (ICU adj2 psychos#s).mp. | 9 |
| 42 | (Intensive Care adj3 psychos#s).mp. | 9 |
| 43 | (metabolic adj1 encephalopath*).mp. | 107 |
| 44 | pseudosenilit*.mp. | 0 |
| 45 | pseudo-senilit*.mp. | 0 |
| 46 | (reversab* adj2 dementia?).mp. | 0 |
| 47 | (reversib* adj2 dementia?).mp. | 155 |
| 48 | (toxic adj2 psychos#s).mp. | 343 |
| 49 | or/1-48 [ Delirium & related terms ] | 602301 |
| 50 | Cognitive Assessment/ | 4127 |
| 51 | Algorithms/ | 16926 |
| 52 | Data Collection/ | 6347 |
| 53 | exp "checklist (testing)"/ | 3843 |
| 54 | comprehension tests/ | 200 |
| 55 | geriatric assessment/ | 929 |
| 56 | individual testing/ | 168 |
| 57 | exp inventories/ | 10110 |
| 58 | exp perceptual measures/ | 1780 |
| 59 | exp physical health assessment/ | 11702 |
| 60 | exp psychological assessment/ | 40588 |
| 61 | psychometrics/ | 55730 |
| 62 | exp questionnaires/ | 17480 |
| 63 | "Quality of Life"/ | 36902 |
| 64 | exp rating scales/ | 21680 |
| 65 | screening/ | 9143 |
| 66 | screening tests/ | 5444 |
| 67 | standardized tests/ | 1446 |
| 68 | surveys/ | 7568 |
| 69 | exp symptom checklists/ | 749 |
| 70 | exp test battery/ | 2139 |
| 71 | exp testing methods/ | 7294 |
| 72 | algorithm*.mp. | 31344 |
| 73 | assess*.mp. | 745170 |
| 74 | audit?.mp. | 6913 |
| 75 | (battery or batteries).mp. | 48551 |
| 76 | checklist?.mp. | 61788 |
| 77 | check-list?.mp. | 5221 |
| 78 | evaluat*.mp. | 527942 |
| 79 | exam*.mp. | 1138715 |
| 80 | focus group*.mp. | 31009 |
| 81 | (health adj2 determinant*).mp. | 2958 |
| 82 | HRQoL.mp. | 3976 |
| 83 | index*.mp. | 191603 |
| 84 | indices.mp. | 28972 |
| 85 | indicator?.mp. | 62521 |
| 86 | interview*.mp. | 360218 |
| 87 | instrument?.mp. | 109164 |
| 88 | inventories.mp. | 16220 |
| 89 | inventory.mp. | 216108 |
| 90 | measur*.mp. | 807811 |
| 91 | metric?.mp. | 11878 |
| 92 | narrati*.mp. | 62138 |
| 93 | nomogram*3.mp. | 151 |
| 94 | patient analys#s.mp. | 75 |
| 95 | poll?.mp. | 2502 |
| 96 | QoL.mp. | 8950 |
| 97 | quality of life.mp. | 68119 |
| 98 | questionnaire?.mp. | 366825 |
| 99 | rating?.mp. | 208985 |
| 100 | (scale or scales).mp. | 610613 |
| 101 | screening?.mp. | 73289 |
| 102 | (score or scores or scored or scoring?).mp. | 323107 |
| 103 | status exam*.mp. | 2634 |
| 104 | survey*.mp. | 298876 |
| 105 | (test or tests).mp. | 657082 |
| 106 | (tool? or toolkit? or tool-kit?).mp. | 137209 |
| 107 | "diagnostic and statistical manual of mental disorders".mp. | 20592 |
| 108 | "diagnostic & statistical manual of mental disorders".mp. | 170 |
| 109 | DSM.mp. | 77906 |
| 110 | (DSM1 or DSM2 or DSM3 or DSM4 or DSM5).mp. | 76 |
| 111 | or/50-110 [ Assessment / Tools / Questionnaires ] | 2903724 |
| 112 | 49 and 111 [ Delirium + Assessment ] | 429166 |
| 113 | (preoperat* or pre-operat*).mp. | 5494 |
| 114 | (before adj2 surg*).mp. | 1598 |
| 115 | (before adj2 procedur*).mp. | 331 |
| 116 | (before adj2 operat????).mp. | 438 |
| 117 | (prior adj2 procedur*).mp. | 158 |
| 118 | (prior adj2 operat????).mp. | 100 |
| 119 | (prior adj1 surg*).mp. | 67 |
| 120 | pre-procedur*.mp. | 37 |
| 121 | preprocedur*.mp. | 80 |
| 122 | preintervention*.mp. | 770 |
| 123 | pre-intervention*.mp. | 1402 |
| 124 | pre-surg*.mp. | 515 |
| 125 | "pre- and post-surgery".mp. | 74 |
| 126 | "pre- and post-surgical".mp. | 52 |
| 127 | "pre- and postsurgery".mp. | 47 |
| 128 | "pre- and postsurgical".mp. | 57 |
| 129 | or/113-118 [ Pre-Operative ] | 7566 |
| 130 | 112 and 129 [ Delirium + Assessment + Pre-operative ] | 1218 |
| 131 | postsurgical complications/ | 821 |
| 132 | "after surgery".mp. | 4643 |
| 133 | "after surgical*".mp. | 538 |
| 134 | (following adj2 (surgery or surgeries or surgical)).mp. | 2115 |
| 135 | (postoperat* or post-operat*).mp. | 9633 |
| 136 | post-intervention*.mp. | 5891 |
| 137 | postintervention*.mp. | 2476 |
| 138 | Post-procedur*.mp. | 96 |
| 139 | postprocedur*.mp. | 82 |
| 140 | post-surg*.mp. | 1273 |
| 141 | postsurg*.mp. | 2296 |
| 142 | or/131-141 [ Postoperative ] | 24456 |
| 143 | 130 and 142 [ Delirium + Assessment + Pre-operative + Postoperative ] | 694 |
| 144 | limit 143 to english language | 638 |
| 145 | limit 144 to (2200 psychometrics & statistics & methodology or 2220 tests & testing or 2221 sensory & motor testing or 2224 clinical psychological testing or 2225 neuropsychological assessment or 2226 health psychology testing or 2340 cognitive processes or 2380 consciousness states or 2520 neuropsychology & neurology or 2540 physiological processes or 3040 social perception & cognition or 3210 psychological disorders or 3290 physical & somatoform & psychogenic disorders or 3299 vision & hearing & sensory disorders or 3360 health psychology & medicine or "3365 promotion & maintenance of health & wellness" or 3379 inpatient & hospital services) | 110 |
| 146 | limit 144 to tests & measures | 26 |
| 147 | 145 or 146 | 110 |
| 148 | limit 147 to ("0200 clinical case study" or "0300 clinical trial" or "0430 followup study" or "0450 longitudinal study" or "0451 prospective study" or "0453 retrospective study" or "0830 systematic review" or 1200 meta analysis or 1300 metasynthesis) | 43 |
| 149 | random*.mp. | 179781 |
| 150 | ((single or double or triple or treble) adj3 (blind* or mask*)).mp. | 24419 |
| 151 | doubleblind*.mp. | 173 |
| 152 | Placebo*.mp. | 38051 |
| 153 | cohort*.mp. | 66676 |
| 154 | (evaluation adj1 (study or studies)).mp. | 2741 |
| 155 | (validation adj1 (study or studies)).mp. | 4622 |
| 156 | (controlled adj1 clinical adj2 (trial? or study or studies)).mp. | 3128 |
| 157 | (meta-anal* or metanal* or metaanal*).mp. | 31448 |
| 158 | (systematic adj4 (review or reviews or overview or overviews)).mp. | 25295 |
| 159 | (overview? adj4 (review or reviews)).mp. | 1349 |
| 160 | (case control* adj2 (study or studies)).mp. | 7380 |
| 161 | (longitudinal* adj2 (study or studies)).mp. | 61600 |
| 162 | (prospective* adj2 (study or studies)).mp. | 24701 |
| 163 | (cross-sectional* adj2 (study or studies)).mp. | 26503 |
| 164 | case series.mp. | 3481 |
| 165 | (retrospective* adj2 (study or studies)).mp. | 10006 |
| 166 | or/149-165 [ Studies ] | 411780 |
| 167 | 147 and 166 | 42 |
| 168 | 148 or 167 | 60 |
| 169 | limit 168 to journal article | 53 |
| 170 | remove duplicates from 169 | 53 |
| 171 | biological markers/ | 10745 |
| 172 | biomarker?.mp. | 14891 |
| 173 | bio-marker?.mp. | 46 |
| 174 | (marker or markers).mp. | 51933 |
| 175 | proteomics/ | 527 |
| 176 | proteomic??.mp. | 1452 |
| 177 | proteogenomic??.mp. | 0 |
| 178 | phenotypes/ | 9749 |
| 179 | endophenotyp???.mp. | 3305 |
| 180 | phenotyp???.mp. | 33572 |
| 181 | or/171-180 [ Biological Markers & related terms ] | 91605 |
| 182 | 49 and 129 and 142 and 181 | 37 |

# PubMed-NOT-Medline

| Search | Query | Items found |
| --- | --- | --- |
| #5 | Search ((((((Emergence Delirium OR Cognition Disorders OR Mild Cognitive Impairment OR Cognition OR Confusion OR Delirium OR Dementia OR Hallucinations OR (cognitiv* AND impair*) OR (disorder* AND cognit*) OR (cognitiv* AND status) OR (cognitiv* AND dysfunc*) OR (cognitiv* AND function*) OR dyscognitiv* OR cognitive* OR cognition* OR confuse OR confused OR confusing OR confusion OR delirium OR delirius OR delirious OR hallucinat* OR dementia OR demented OR dementat* OR (intellectual* AND declin*) OR (mental* AND deteriorat*) OR (mental* AND acuit*) OR (post-anaesth* AND excit*) OR (post-anesth* AND excit*) OR (postanaesth* AND excit*) OR (postanesth* AND excit*) OR (emergence AND agitat*) OR (emergence AND excit*) OR ("acute confusional state") OR ("acute confusional states") OR ("acute confusioned state") OR ("acute confusioned states") OR ("toxic-metabolic encephalopathy") OR ("toxic-metabolic encephalopathies") OR ("acute brain syndrome") OR ("acute brain syndromes") OR ("acute brain failure") OR ("acute organic psychoses") OR ("acute organic psychosis") OR ("acute organic brain syndrome") OR ("acute organic brain syndromes") OR ("ICU psychoses") OR ("ICU psychosis") OR ("intensive care psychoses") OR ("intensive care psychosis") OR ("metabolic encephalopathy") OR ("metabolic encephalopathies") OR (pseudosenilit*) OR (pseudo-senilit*) OR ("reversable dementia") OR ("reversible dementia") OR ("toxic psychoses") OR ("toxic psychosis")))) AND ((Preoperative Care OR preoperative period OR (preoperat* OR pre-operat*) OR preoperative OR pre-operative OR pre-procedure OR pre-procedural OR preprocedure OR preprocedural OR preintervention OR preinterventions OR pre-intervention OR pre-interventions OR pre-surgery OR pre-surgical OR "pre- and post-surgery" OR "pre- and post-surgical" OR "pre- and postsurgery" OR "pre- and postsurgical")))) AND (((Surgical Procedures, Operative OR surgery[MeSH subheading] OR postoperative care OR Postoperative Period OR Postoperative Complications OR "after surgery" OR "after surgical" OR postoperative OR postoperatively OR post-operative OR post-operatively OR post-intervention OR post-interventional OR postintervention OR postinterventional OR post-procedure OR post-procedural OR postprocedure OR postprocedural OR post-surgery OR post-surgical OR postsurgery OR postsurgical))) AND ((Symptom Assessment OR Algorithms OR Data Collection OR Focus Groups OR Health Care Surveys OR Health Impact Assessment OR Health Status Indicators OR Health Surveys OR Interviews As Topic OR Narration OR Patient Acuity OR Patient Reported Outcome Measures OR "Quality of Life" OR "Severity Of Illness Index" OR Sickness Impact Profile OR "Surveys and Questionnaires" OR Psychological Tests OR Trail Making Test OR Psychiatric Status Rating Scales OR Geriatric Assessment OR Mass Screening OR algorithm* OR assess OR assesses OR assessed OR assessing OR assessment OR assessments OR audit OR audits OR audited OR auditing OR (battery OR batteries) OR checklist OR checklists OR check-list OR check-lists OR exam OR exams OR examination OR examinations OR evaluation OR evaluations OR focus group OR focus groups OR (health AND determinant) OR (health AND determinants) OR HRQoL OR Index OR indexes OR indices OR indicator OR indicators OR interview* OR instrument OR instruments OR inventories OR inventory OR measure OR measures OR measurement OR measurements OR metric OR metrics OR narrative OR narratives OR narration OR narrations OR nomogram OR nomograms OR nomogramme OR nomogrammes OR patient analysis OR patient analyses OR poll OR polls OR QoL OR quality of life OR questionnaire OR questionnaires OR rating OR ratings OR (scale or scales) OR screening OR screenings OR (score OR scores OR scored OR scoring OR scoring) OR status examination OR status examinations OR status exam OR status exams OR survey OR surveyed OR surveying OR surveys OR test OR tests OR (tool OR tools OR toolkit OR toolkits OR tool-kit OR tool-kits) OR DSM OR ("diagnostic and statistical manual of mental disorders") OR ("diagnostic & statistical manual of mental disorders")))) AND ((Biomarkers OR biomarker OR bio-marker OR bio-markers OR marker OR markers OR Proteomics OR Proteogenomics OR proteomic* OR proteogenomic* OR Endophenotypes OR endophenotyp* or Genetic Markers))) AND (((Randomized controlled trial OR Randomized controlled trials OR Randomized Controlled Trials as Topic OR Pragmatic Clinical Trial OR Pragmatic Clinical Trials OR Pragmatic Clinical Trials As Topic OR Double-Blind Method OR Placebos OR placebo OR clinical trial OR clinical trials OR Controlled Clinical Trial OR Controlled Clinical Trials OR Controlled Clinical Trials As Topic OR meta-analysis OR meta-analyses OR meta-analysis as topic OR Cohort Studies OR Cohort study OR Case-Control Studies OR Case-control study OR Longitudinal Studies OR Longitudinal study OR Prospective Studies OR Prospective study OR Cross-Sectional Studies OR Cross-sectional study OR Retrospective Studies OR Retrospective study OR evaluation studies OR evaluation study OR validation studies OR validation study OR "sensitivity and specificity" OR "predictive value of tests" OR roc curve OR roc curves OR random* OR doubleblind* OR double-blind* OR cohort OR cohorts OR case series OR (meta-anal* OR metanal* OR metaanal*) OR (systematic AND (review OR reviews OR overview OR overviews)) OR (overview AND (review OR reviews)) OR (overviews AND (review OR reviews)) OR (case control AND (study OR studies)) OR (longitudinal AND (study OR studies)) OR (prospective AND (study OR studies)) OR (cross-sectional AND (study OR studies)) OR (retrospective AND (study OR studies))) NOT Medline[sb] Sort by: PublicationDate | 4 |

# CINAHL

Preop Assess Postop Delirium Biomarkers - REVISED

Top of Form

| **#** | **Query** | **Limiters/Expanders** | **Last Run Via** | **Results** |
| --- | --- | --- | --- | --- |
| S39 | S30 AND S37 | Limiters - Peer Reviewed  Search modes - Boolean/Phrase | Interface - EBSCOhost Research Databases  Search Screen - Advanced Search  Database - CINAHL with Full Text | 6 |
| S38 | S30 AND S37 | Search modes - Boolean/Phrase | Interface - EBSCOhost Research Databases  Search Screen - Advanced Search  Database - CINAHL with Full Text | 7 |
| S37 | S31 OR S32 OR S33 OR S34 OR S35 OR S36 | Search modes - Boolean/Phrase | Interface - EBSCOhost Research Databases  Search Screen - Advanced Search  Database - CINAHL with Full Text | 69,057 |
| S36 | (MH "Genetic Markers") | Search modes - Boolean/Phrase | Interface - EBSCOhost Research Databases  Search Screen - Advanced Search  Database - CINAHL with Full Text | 1,610 |
| S35 | endophenotyp* | Search modes - Boolean/Phrase | Interface - EBSCOhost Research Databases  Search Screen - Advanced Search  Database - CINAHL with Full Text | 268 |
| S34 | (proteomic* OR proteogenomic*) | Search modes - Boolean/Phrase | Interface - EBSCOhost Research Databases  Search Screen - Advanced Search  Database - CINAHL with Full Text | 1,908 |
| S33 | (marker OR markers) | Search modes - Boolean/Phrase | Interface - EBSCOhost Research Databases  Search Screen - Advanced Search  Database - CINAHL with Full Text | 57,557 |
| S32 | (biomarker* OR bio-maker*) | Search modes - Boolean/Phrase | Interface - EBSCOhost Research Databases  Search Screen - Advanced Search  Database - CINAHL with Full Text | 16,326 |
| S31 | (MH "Biological Markers+") | Search modes - Boolean/Phrase | Interface - EBSCOhost Research Databases  Search Screen - Advanced Search  Database - CINAHL with Full Text | 33,533 |
| S30 | S21 AND S28 | Limiters - Exclude MEDLINE records  Search modes - Boolean/Phrase | Interface - EBSCOhost Research Databases  Search Screen - Advanced Search  Database - CINAHL with Full Text | 68 |
| S29 | S21 AND S28 | Search modes - Boolean/Phrase | Interface - EBSCOhost Research Databases  Search Screen - Advanced Search  Database - CINAHL with Full Text | 273 |
| S28 | S22 OR S23 OR S24 OR S25 OR S26 OR S27 | Search modes - Boolean/Phrase | Interface - EBSCOhost Research Databases  Search Screen - Advanced Search  Database - CINAHL with Full Text | 1,407,251 |
| S27 | (status N2 exam*) OR ( battery OR batteries ) OR (quality N2 life) OR ("diagnostic and statistical manual of mental disorders") OR ("diagnostic & statistical manual of mental disorders") OR DSM | Search modes - Boolean/Phrase | Interface - EBSCOhost Research Databases  Search Screen - Advanced Search  Database - CINAHL with Full Text | 110,050 |
| S26 | (interview* OR instrument* OR tool* OR toolkit* ) OR ( measur* OR metric* OR rating* OR scale* ) OR ( screen* OR score* OR survey* ) OR exam* OR test OR tests | Search modes - Boolean/Phrase | Interface - EBSCOhost Research Databases  Search Screen - Advanced Search  Database - CINAHL with Full Text | 1,301,498 |
| S25 | algorithm* OR check-list* OR checklist* | Search modes - Boolean/Phrase | Interface - EBSCOhost Research Databases  Search Screen - Advanced Search  Database - CINAHL with Full Text | 43,571 |
| S24 | (MM "Algorithms") OR (MM "Decision Trees") | Search modes - Boolean/Phrase | Interface - EBSCOhost Research Databases  Search Screen - Advanced Search  Database - CINAHL with Full Text | 2,768 |
| S23 | (MH "Clinical Assessment Tools+") OR (MH "Instrument by Type") OR (MH "Interview Guides+") OR (MH "Psychological Tests+") OR (MH "Questionnaires+") OR (MH "Scales") OR (MH "Videorecording") OR (MH "Behavior Rating Scales") OR (MH "Checklists") OR (MH "Daily Logs") OR (MH "Diaries") OR (MH "Language Tests+") | Search modes - Boolean/Phrase | Interface - EBSCOhost Research Databases  Search Screen - Advanced Search  Database - CINAHL with Full Text | 487,089 |
| S22 | (MH "Patient Assessment+") OR (MH "Geriatric Functional Assessment") OR (MH "Clinical Assessment Tools+") | Search modes - Boolean/Phrase | Interface - EBSCOhost Research Databases  Search Screen - Advanced Search  Database - CINAHL with Full Text | 177,241 |
| S21 | S3 AND S6 AND S20 | Search modes - Boolean/Phrase | Interface - EBSCOhost Research Databases  Search Screen - Advanced Search  Database - CINAHL with Full Text | 318 |
| S20 | S7 OR S8 OR S9 OR S10 OR S11 OR S12 OR S13 OR S14 OR S15 OR S16 OR S17 OR S18 OR S19 | Search modes - Boolean/Phrase | Interface - EBSCOhost Research Databases  Search Screen - Advanced Search  Database - CINAHL with Full Text | 35,262 |
| S19 | (emergence N1 agitat*) | Search modes - Boolean/Phrase | Interface - EBSCOhost Research Databases  Search Screen - Advanced Search  Database - CINAHL with Full Text | 37 |
| S18 | (post-anesth* N1 excit*) OR (post-anaesth* N1 excit*) OR (postanesth* N1 excit*) OR (postanaesth* N1 excit*) | Search modes - Boolean/Phrase | Interface - EBSCOhost Research Databases  Search Screen - Advanced Search  Database - CINAHL with Full Text | 1 |
| S17 | (mental* N2 acuit*) | Search modes - Boolean/Phrase | Interface - EBSCOhost Research Databases  Search Screen - Advanced Search  Database - CINAHL with Full Text | 44 |
| S16 | (mental* N2 deteriorat*) | Search modes - Boolean/Phrase | Interface - EBSCOhost Research Databases  Search Screen - Advanced Search  Database - CINAHL with Full Text | 165 |
| S15 | (intellectual* N2 declin*). | Search modes - Boolean/Phrase | Interface - EBSCOhost Research Databases  Search Screen - Advanced Search  Database - CINAHL with Full Text | 39 |
| S14 | confus* | Search modes - Boolean/Phrase | Interface - EBSCOhost Research Databases  Search Screen - Advanced Search  Database - CINAHL with Full Text | 11,035 |
| S13 | (MH "Confusion+") OR (MH "Confusion (Saba CCC)") OR (MH "Acute Confusion (NANDA)") OR (MH "Confusion Management (Iowa NIC)") | Search modes - Boolean/Phrase | Interface - EBSCOhost Research Databases  Search Screen - Advanced Search  Database - CINAHL with Full Text | 5,100 |
| S12 | hallucinat* | Search modes - Boolean/Phrase | Interface - EBSCOhost Research Databases  Search Screen - Advanced Search  Database - CINAHL with Full Text | 2,223 |
| S11 | (MH "Hallucinations") OR (MH "Hallucination Management (Iowa NIC)") | Search modes - Boolean/Phrase | Interface - EBSCOhost Research Databases  Search Screen - Advanced Search  Database - CINAHL with Full Text | 1,341 |
| S10 | (cogniti* N2 dysfunc*) OR (acute confusional state*) OR (toxic-metabolic N1 encephalopath*) OR (acute brain N1 syndrome*) OR (acute brain N1 fail*) OR (acute organic psycho*) OR (acute organic brain*) OR (ICU N1 psycho*) OR (intensive care N1 psycho*) OR (metabolic N1 encephalopath*) OR (pseudosenilit*) OR (pseudo-senilit*) OR (reversab* N2 dementia*) OR (reversib* N2 dementia*) OR (toxic N2 psycho*) | Search modes - Boolean/Phrase | Interface - EBSCOhost Research Databases  Search Screen - Advanced Search  Database - CINAHL with Full Text | 2,599 |
| S9 | (MH "Cognition Disorders+") | Search modes - Boolean/Phrase | Interface - EBSCOhost Research Databases  Search Screen - Advanced Search  Database - CINAHL with Full Text | 16,521 |
| S8 | deliri* | Search modes - Boolean/Phrase | Interface - EBSCOhost Research Databases  Search Screen - Advanced Search  Database - CINAHL with Full Text | 5,626 |
| S7 | (MH "Delirium") OR (MH "Delirium, Dementia, Amnestic, Cognitive Disorders") OR (MH "Delirium Management (Iowa NIC)") | Search modes - Boolean/Phrase | Interface - EBSCOhost Research Databases  Search Screen - Advanced Search  Database - CINAHL with Full Text | 3,944 |
| S6 | S4 OR S5 | Search modes - Boolean/Phrase | Interface - EBSCOhost Research Databases  Search Screen - Advanced Search  Database - CINAHL with Full Text | 31,904 |
| S5 | preoperat* OR pre-operat* | Search modes - Boolean/Phrase | Interface - EBSCOhost Research Databases  Search Screen - Advanced Search  Database - CINAHL with Full Text | 29,745 |
| S4 | (MH "Preoperative Care+") | Search modes - Boolean/Phrase | Interface - EBSCOhost Research Databases  Search Screen - Advanced Search  Database - CINAHL with Full Text | 12,694 |
| S3 | S1 OR S2 | Search modes - Boolean/Phrase | Interface - EBSCOhost Research Databases  Search Screen - Advanced Search  Database - CINAHL with Full Text | 85,628 |
| S2 | postoperat* OR post-operat* | Search modes - Boolean/Phrase | Interface - EBSCOhost Research Databases  Search Screen - Advanced Search  Database - CINAHL with Full Text | 74,012 |
| S1 | (MH "Postoperative Care+") OR (MH "Postoperative Period") OR (MH "Postoperative Complications+") OR (MH "Postoperative Pain") OR (MH "Postoperative Hemorrhage") | Search modes - Boolean/Phrase | Interface - EBSCOhost Research Databases  Search Screen - Advanced Search  Database - CINAHL with Full Text | 62,330 |

Bottom of Form

# Web of Science

Preop Postop Delir B

| History Name: | Preop Postop Delir B |
| --- | --- |
| Description | Preop Assess postop delirium biomarker U |
| Query: | (#8 NOT #9) *AND* **LANGUAGE:** (English) |

Web of Science Core Collection: Citation Indexes

Science Citation Index Expanded (SCI-EXPANDED) --1900-present

Social Sciences Citation Index (SSCI) --1956-present

Arts & Humanities Citation Index (A&HCI) --1975-present

Conference Proceedings Citation Index- Science (CPCI-S) --1990-present

Conference Proceedings Citation Index- Social Science & Humanities (CPCI-SSH) --1990-present

Emerging Sources Citation Index (ESCI) --2015-present

Data last updated: 2018-06-21

|  | Results | Query |
| --- | --- | --- |
| # 10 | 2 | (#8 NOT #9) *AND* **LANGUAGE:** (English)  Indexes=SCI-EXPANDED, SSCI, A&HCI Timespan=All years |
| # 9 | 18,933,176 | ((PMID=(0* OR 1* OR 2* OR 3* OR 4* OR 5* OR 6* OR 7* OR 8* OR 9*))) *AND* **LANGUAGE:** (English)  Indexes=SCI-EXPANDED, SSCI, A&HCI Timespan=All years |
| # 8 | 97 | #7 AND #5 AND #4 AND #3 AND #1  Indexes=SCI-EXPANDED, SSCI, A&HCI Timespan=All years |
| # 7 | 1,214,314 | (TS=(biomarker* OR bio-marker* OR marker OR markers OR proteomic* OR proteogenomic* OR phenotyp* OR endophenotyp*)) *AND* **LANGUAGE:** (English) *AND* **DOCUMENT TYPES:** (Article)  Indexes=SCI-EXPANDED, SSCI, A&HCI Timespan=All years |
| # 6 | 1,572 | #5 AND #4 AND #3 AND #2 AND #1 s  Indexes=SCI-EXPANDED, SSCI, A&HCI Timespan=All years |
| # 5 | 3,362,254 | (TS=(Randomized controlled trial OR Randomized controlled trials OR Randomized Controlled Trials as Topic OR Pragmatic Clinical Trial OR Pragmatic Clinical Trials OR Pragmatic Clinical Trials As Topic OR Double-Blind Method OR Placebos OR placebo OR clinical trial OR clinical trials OR Controlled Clinical Trial OR Controlled Clinical Trials OR Controlled Clinical Trials As Topic OR meta-analysis OR meta-analyses OR meta-analysis as topic OR Cohort Studies OR Cohort study OR Case-Control Studies OR Case-control study OR Longitudinal Studies OR Longitudinal study OR Prospective Studies OR Prospective study OR Cross-Sectional Studies OR Cross-sectional study OR Retrospective Studies OR Retrospective study OR evaluation studies OR evaluation study OR validation studies OR validation study OR "sensitivity and specificity" OR "predictive value of tests" OR roc curve OR roc curves OR random* OR doubleblind* OR double-blind* OR cohort OR cohorts OR case series OR (meta-anal* OR metanal* OR metaanal*) OR (systematic NEAR/3 (review OR reviews OR overview OR overviews)) OR (overview NEAR/3 review*) OR (overviews NEAR/3 review*) OR (case control NEAR/3 stud*) OR (longitudinal NEAR/3 stud*) OR (prospective NEAR/3 stud*) OR (cross-sectional NEAR/3 stud*) OR (retrospective NEAR/3 stud*))) *AND* **LANGUAGE:** (English) *AND* **DOCUMENT TYPES:** (Article)  Indexes=SCI-EXPANDED, SSCI, A&HCI Timespan=All years |
| # 4 | 854,555 | (TS=(Surgical Procedures, Operative OR surgery OR postoperative care OR Postoperative Period OR Postoperative Complications OR "after surgery" OR "after surgical" OR postoperative OR postoperatively OR post-operative OR post-operatively OR post-intervention OR post-interventional OR postintervention OR postinterventional OR post-procedure OR post-procedural OR postprocedure OR postprocedural OR post-surgery OR post-surgical OR postsurgery OR postsurgical)) *AND* **LANGUAGE:** (English) *AND* **DOCUMENT TYPES:** (Article)  Indexes=SCI-EXPANDED, SSCI, A&HCI Timespan=All years |
| # 3 | 191,487 | (TS=(Preoperative Care OR preoperative period OR (preoperat* OR pre-operat*) OR preoperative OR pre-operative OR pre-procedure OR pre-procedural OR preprocedure OR preprocedural OR preintervention OR preinterventions OR pre-intervention OR pre-interventions OR pre-surgery OR pre-surgical OR "pre- and post-surgery" OR "pre- and post-surgical" OR "pre- and postsurgery" OR "pre- and postsurgical")) *AND* **LANGUAGE:** (English) *AND* **DOCUMENT TYPES:** (Article)  Indexes=SCI-EXPANDED, SSCI, A&HCI Timespan=All years |
| # 2 | 17,693,229 | (TS=(Symptom Assessment OR Algorithms OR Data Collection OR Focus Groups OR Health Care Surveys OR Health Impact Assessment OR Health Status Indicators OR Health Surveys OR Interviews As Topic OR Narration OR Patient Acuity OR Patient Reported Outcome Measures OR "Quality of Life" OR "Severity Of Illness Index" OR Sickness Impact Profile OR "Surveys and Questionnaires" OR Psychological Tests OR Trail Making Test OR Psychiatric Status Rating Scales OR Geriatric Assessment OR Mass Screening OR algorithm* OR assess OR assesses OR assessed OR assessing OR assessment OR assessments OR audit OR audits OR audited OR auditing OR (battery OR batteries) OR checklist OR checklists OR check-list OR check-lists OR evaluation OR evaluations OR exam* OR focus group OR focus groups OR (health NEAR/3 determinant) OR (health NEAR/3 determinants) OR HRQoL OR Index OR indexes OR indices OR indicator OR indicators OR interview* OR instrument OR instruments OR inventories OR inventory OR measure OR measures OR measurement OR measurements OR metric OR metrics OR narrative OR narratives OR narration OR narrations OR nomogram OR nomograms OR nomogramme OR nomogrammes OR patient analysis OR patient analyses OR poll OR polls OR QoL OR quality of life OR questionnaire OR questionnaires OR rating OR ratings OR (scale or scales) OR screening OR screenings OR (score OR scores OR scored OR scoring OR scoring) OR status examination OR status examinations OR status exam OR status exams OR survey OR surveyed OR surveying OR surveys OR test OR tests OR (tool OR tools OR toolkit OR toolkits OR tool-kit OR tool-kits) OR DSM OR ("diagnostic and statistical manual of mental disorders") OR ("diagnostic & statistical manual of mental disorders"))) *AND* **LANGUAGE:** (English)  Indexes=SCI-EXPANDED, SSCI, A&HCI Timespan=All years |
| # 1 | 655,153 | (TS=(Emergence Delirium OR Cognition Disorders OR Mild Cognitive Impairment OR Cognition OR Confusion OR Delirium OR Dementia OR Hallucinations OR (cognitiv* NEAR/2 impair*) OR (disorder* NEAR/3 cognit*) OR (cognitiv* NEAR/3 status) OR (cognitiv* NEAR/3 dysfunc*) OR (cognitiv* NEAR/3 function*) OR dyscognitiv* OR cognitive* OR cognition* OR confuse OR confused OR confusing OR confusion OR delirium OR delirius OR delirious OR hallucinat* OR dementia OR demented OR dementat* OR (intellectual* NEAR/2 declin*) OR (mental* NEAR/2 deteriorat*) OR (mental* NEAR/2 acuit*) OR (post-anaesth* NEAR/2 excit*) OR (post-anesth* NEAR/2 excit*) OR (postanaesth* NEAR/2 excit*) OR (postanesth* AND excit*) OR (emergence NEAR/2 agitat*) OR (emergence NEAR/3 excit*) OR (acute confusion* state*) OR (toxic-metabolic encephalopath*) OR (acute brain syndrome*) OR (acute brain fail*) OR (acute organ* psychosis) OR (acute organ* psychoses) OR (acute organ* brain) OR (ICU NEAR/2 psychosis) OR (ICU NEAR/2 psychoses) OR (intensive care NEAR/3 psychoses) OR (intensive care NEAR/3 psychosis) OR (metabolic NEAR/1 encephalopath*) OR pseudosenilit* OR pseudo-senilit* OR (revers* NEAR/2 dementia*) OR (toxic NEAR/2 psycho*))) *AND* **LANGUAGE:** (English)  Indexes=SCI-EXPANDED, SSCI, A&HCI Timespan=All years |

# SCOPUS

Preop Assess Postop Delirium and Biomarkers - REVISED June 22 2018

# 6 document results

( TITLE-ABS-KEY ( biomarker*  OR  bio-marker*  OR  marker  OR  markers  OR  proteomic*  OR  proteogenomic*  OR  phenotyp*  OR  endophenotyp* )  AND  ( TITLE-ABS-KEY ( ( ( emergence  W/1  delirium )  OR  ( cognition  AND  disorders )  OR  ( mild  AND  cognitive  AND  impairment )  OR  cognition  OR  confusion  OR  delirium  OR  dementia  OR  hallucinations  OR  ( cognitiv*  W/2  impair* )  OR  ( disorder*  W/3  cognit* )  OR  ( cognitiv*  W/3  status )  OR  ( cognitiv*  W/3  dysfunc* )  OR  ( cognitiv*  W/3  function* )  OR  dyscognitiv*  OR  cognitive*  OR  cognition*  OR  confuse  OR  confused  OR  confusing  OR  confusion  OR  delirium  OR  delirius  OR  delirious  OR  hallucinat*  OR  dementia  OR  demented  OR  dementat*  OR  ( intellectual*  W/2  declin* )  OR  ( mental*  W/2  deteriorat* )  OR  ( mental*  W/2  acuit* )  OR  ( post-anaesth*  W/2  excit* )  OR  ( post-anesth*  W/2  excit* )  OR  ( postanaesth*  W/2  excit* )  OR  ( postanesth*  AND  excit* )  OR  ( emergence  W/2  agitat* )  OR  ( emergence  W/3  excit* )  OR  ( acute  AND confusion* )  OR  ( toxic-metabolic  AND encephalopath* )  OR  ( acute  AND brain  AND syndrome* )  OR  ( acute  AND brain  AND fail* )  OR  ( acute  AND organic  AND psycho* )  OR  ( acute  AND organic  AND brain )  OR  ( icu  AND psycho* )  OR  ( intensive  AND care  AND psycho* )  OR  ( metabolic  AND encephalopath* )  OR  ( pseudosenil* )  OR  ( pseudo-senil* )  OR  ( revers*  W/2  dement* )  OR  ( toxic  AND psycho* ) ) ) )  AND  ( TITLE-ABS-KEY ( ( ( preoperative  W/2  care )  OR  ( preoperative  W/2  period* )  OR  preoperat*  OR  pre-operat*  OR  preoperative  OR  pre-operative  OR  pre-procedure  OR  pre-procedural  OR  preprocedure  OR  preprocedural  OR  preintervention  OR  preinterventions  OR  pre-intervention  OR  pre-interventions  OR  pre-surgery  OR  pre-surgical  OR  "pre- and post-surgery"  OR  "pre- and post-surgical"  OR  "pre- and postsurgery"  OR  "pre- and postsurgical" ) ) )  AND  ( TITLE-ABS-KEY ( ( surgical  W/1  procedure* )  OR  surgery  OR  ( postoperative  W/1  care )  OR  ( postoperative  W/1  period* )  OR  ( postoperative  W/1  complication* )  OR  "after surgery"  OR  "after surgical"  OR  postoperative  OR  postoperatively  OR  post-operative  OR  post-operatively  OR  post-intervention  OR  post-interventional  OR  postintervention  OR  postinterventional  OR  post-procedure  OR  post-procedural  OR  postprocedure  OR  postprocedural  OR  post-surgery  OR  post-surgical  OR  postsurgery  OR  postsurgical ) )  AND  ( TITLE-ABS-KEY ( ( randomized  AND  controlled  AND  trial* )  OR  ( pragmatic  AND  clinical  AND  trial* )  OR  ( double-blind  W/1  method* )  OR  placebos  OR  placebo  OR  ( clinical  W/1  trial* )  OR  meta-analysis  OR  meta-analyses  OR  ( cohort  AND  studies )  OR  ( cohort  AND  study )  OR  ( case-control  AND  studies )  OR  ( case-control  AND  study )  OR  ( longitudinal  W/1  stu* )  OR  ( prospective  W/1  stud* )  ( cross-sectional  W/1  stud* )  OR  ( retrospective  W/1  stud* )  OR  ( evaluation  W/1  stud* )  OR  ( validation  W/1  stud* )  OR  "sensitivity and specificity"  OR  "predictive value of tests"  OR  "roc curve"  OR  "roc curves"  OR  random*  OR  doubleblind*  OR  double-blind*  OR  cohort  OR  cohorts  OR  case  AND  series  OR  ( meta-anal*  OR  metanal*  OR  metaanal* )  OR  ( systematic  W/3  ( review  OR  reviews  OR  overview  OR  overviews ) )  OR  ( overview  W/3  review* )  OR  ( overviews  W/3  review* )  OR  ( case  AND  control  W/3  stud* )  OR  ( longitudinal  W/3  stud* )  OR  ( prospective  W/3  stud* )  OR  ( cross-sectional  W/3  stud* )  OR  ( retrospective  W/3  stud* ) ) ) )  AND NOT  ( PMID ( ( 0*  OR  1*  OR  2*  OR  3*  OR  4*  OR  5*  OR  6*  OR  7*  OR  8*  OR  9* ) ) )  AND  ( LIMIT-TO ( DOCTYPE ,  "ar " )  OR  LIMIT-TO ( DOCTYPE ,  " ip " )  OR  LIMIT-TO ( DOCTYPE ,  " er " ) )  AND  ( LIMIT-TO ( LANGUAGE ,  "English " ) )

# ClinicalTrials.Gov

23 Studies found for: **biomarker OR biomarkers OR marker OR markers | Active, not recruiting, Completed, Suspended, Terminated, Withdrawn, Unknown status Studies | Delirium, Emergence OR postoperative delirium OR Postoperative Cognitive Dysfunction**

Applied Filters: **Active not recruiting Completed Suspended Terminated Withdrawn Unknown status**

# WHO ICTRP

15 records for 15 trials found for: delirium AND biomarkers

Synonyms used:
delirium, Acute brain syndrome, Acute brain syndrome NOS, Acute confusional state, ALCOHOL ABUSE, Brain syndrome acute, Deliria, Delirious, Delirious (finding), SYNDROME BRAIN ACUTE AND biomarker, BIOL MARKER, BIOL MARKERS, Biologic Marker, Biologic Markers, Biological Marker, Biological Markers, marker, MARKER BIOL, Marker Biologic, Marker Biological, MARKERS BIOL, Markers Biologic, Markers Biological, molecular marker, signature molecule
