# Supplementary material for: Pre-operative biomarkers and imaging tests as predictors of post-operative delirium in non-cardiac surgical patients: a systematic review
Source: BMC Anesthesiol. 2019 Feb 23;19:25. doi: 10.1186/s12871-019-0693-y (PMC6387490; doi:10.1186/s12871-019-0693-y)
Supplement: Supplementary file 2 — Supplementary Table S1. QUIPS Risk of bias assessment.. (DOCX 88 kb) [file 12871_2019_693_MOESM2_ESM.docx]

**Supplementary table:** QUIPS Risk of bias assessment.

| Study | Study Participation | Study Attrition | Prognostic Factor Measurement | Outcome Measurement | Confounding  measurement | Statistical Analysis |
| --- | --- | --- | --- | --- | --- | --- |
| Lemstra et al. 2008 (12) | L | L | L | L | M | L |
| Cerejeira et al. 2011 (14) | L | L | L | L | L | L |
| Witlox et al. 2011 (31) | L | M | L | L | M | L |
| Cerejeira et al. 2012 (15) | M | L | L | L | L | L |
| Leung et al. 2007 (18) | L | L | L | L | L | L |
| Westhoff et al. 2013 (7) | M | L | L | L | M | L |
| Capri et al. 2014 (6) | L | L | L | L | L | L |
| Chen et al. 2014 (16) | L | M | L | L | L | L |
| Watne et al. 2014 (26) | L | L | L | L | L | L |
| Torbergsen et al. 2015 (20) | L | L | L | L | M | L |
| Vasunilashorn et al. 2015 (19) | L | L | L | L | L | L |
| Westhoff et al. 2015 (30) | M | M | L | L | L | L |
| Cavallari et al. 2016 (32) | L | L | L | L | L | L |
| Hall et al. 2016 (24) | L | L | L | L | L | L |
| Hov et al. 2016 (25) | L | L | L | L | L | L |
| Neerland et al. 2016 (9) | L | L | L | L | M | L |
| Scholtens et al. 2016 (5) | L | L | L | L | L | L |
| Shen et al. 2016 (13) | L | L | L | L | L | L |
| Watne et al. 2016 (27) | L | L | L | L | M | L |
| Yen et al. 2016 (35) | L | L | L | L | M | L |
| Cunningham et al. 2017 (17) | L | L | L | L | L | L |
| Dillon et al. 2017 (8) | L | L | L | L | L | L |
| Hshieh et al. 2017 (33) | L | L | L | L | L | L |
| Miller et al. 2017 (36) | H | L | L | H | L | L |
| Hall et al. 2013 (29) | L | M | L | L | M | L |
| Hov et al. 2017 (28) | L | L | L | L | L | L |
| Scholtens et al. 2017 (21) | M | L | L | L | L | L |
| Vasunilashorn et al. 2017 (10) | L | L | L | L | L | L |
| Wyrobek et al. 2017 (22) | L | L | L | L | L | L |
| Xiang et al. 2017 (11) | L | L | L | L | L | L |
| Zhang et al. 2017 (23) | L | L | L | L | L | L |
| Bohner et al. 2003 (34) | M | M | L | L | L | L |
| Racine et al. 2017 (38) | L | L | L | L | L | L |
| Root et al. 2013 (37) | M | L | L | L | L | L |

H, high; M, medium; L, low bias
